# Electronic Skin from High-Throughput Fabrication of Intrinsically Stretchable Lead Zirconate Titanate Elastomer

**DOI:** 10.34133/2020/1085417

**Published:** 2020-10-17

**Authors:** Yiming Liu, Huanxi Zheng, Ling Zhao, Shiyuan Liu, Kuanming Yao, Dengfeng Li, Chunki Yiu, Shenghan Gao, Raudel Avila, Pakpong Chirarattananon, Lingqian Chang, Zuankai Wang, Xian Huang, Zhaoqian Xie, Zhengbao Yang, Xinge Yu

**Affiliations:** ^1^Department of Biomedical Engineering, City University of Hong Kong, Hong Kong 999077, China; ^2^Department of Mechanical Engineering, City University of Hong Kong, Hong Kong 999077, China; ^3^Department of Biomedical Engineering, Tianjin University, Tianjin 300000, China; ^4^Department of Mechanical Engineering, McCormick School of Engineering, Northwestern University, Evanston, IL 60208, USA; ^5^School of Biology Science and Medical Engineering, Beihang University, Beijing 100191, China; ^6^State Key Laboratory of Structural Analysis for Industrial Equipment, Department of Engineering Mechanics, International Research Center for Computational Mechanics, Dalian University of Technology, Dalian 116024, China

## Abstract

Electronic skin made of thin, soft, stretchable devices that can mimic the human skin and reconstruct the tactile sensation and perception offers great opportunities for prosthesis sensing, robotics controlling, and human-machine interfaces. Advanced materials and mechanics engineering of thin film devices has proven to be an efficient route to enable and enhance flexibility and stretchability of various electronic skins; however, the density of devices is still low owing to the limitation in existing fabrication techniques. Here, we report a high-throughput one-step process to fabricate large tactile sensing arrays with a sensor density of 25 sensors/cm^2^ for electronic skin, where the sensors are based on intrinsically stretchable piezoelectric lead zirconate titanate (PZT) elastomer. The PZT elastomer sensor arrays with great uniformity and passive-driven manner enable high-resolution tactile sensing, simplify the data acquisition process, and lower the manufacturing cost. The high-throughput fabrication process provides a general platform for integrating intrinsically stretchable materials into large area, high device density soft electronics for the next-generation electronic skin.

## 1. Introduction

Wearable electronics have attracted great attention around the world in the past decades due to their promising application in health monitoring and human-machine interfaces [1–10]. Skin-like wearable electronics, also known as epidermal electronics that involves advanced material science and structural designs [11–18], exhibits excellent stretchability and multifunctionality and therefore allows creating electronic skin (e-skin) for the sensation of pressure [19–23], humidity [24–27], and temperature [28–32]. Among various sensing capabilities of e-skin, tactile sensing is always the most important part, as which can mimic the basic sensation of skin. To date, many kinds of flexible tactile sensors have been developed based on different working principles, including piezoelectricity, triboelectricity, piezoresistivity, and capacitance; however, challenges remain [19, 21, 33–37]. For instance, piezoresistivity and capacitance-type tactile sensors require external power sources, which complicate the integration and may also increase the device size and weight [38]. Self-powered tactile sensors based on triboelectric devices have been a growing interest for wearables and implantable electronics [39–43]. However, due to the contact-separation working principle in triboelectric electronics, the thickness and stretchability of triboelectric-based flexible tactile sensors are still difficult to meet the requirements for e-skin [44]. Meanwhile, signal crosstalk is also a challenge for triboelectric-based large-scale tactile sensor arrays [45]. Another type of self-powered tactile sensor, piezoelectric-based ones, has been widely reported by using chemical/physical stable inorganic materials, lead zirconate titanate (PZT), which involves a large amount work of structural designs, fabrication, and unconventional processing methods, such as strong acid etching and transfer printing [2, 46, 47]. The multiple step fabrication and unconventional processing methods cause a cost increase, thereby limiting future applications in large-area e-skin. Therefore, developing intrinsically stretchable tactile sensors with a self-powering nature is the key point for a large-area, low-cost, and high-performance e-skin.

Here, we present material design and integration scheme as a simple and efficient approach for tactile e-skin with high density of sensing pixels. The reported e-skin exploits intrinsically stretchable piezoelectric elastomer as sensing pixels by blending PZT nanoparticles with PDMS. One-step screen printing of the piezoelectric elastomer on the preformed in plane electrode-coated soft substrate forms e-skin with high-density sensing pixels. Compared to the conventional sandwich-structured PZT sensors, the in-plane PZT sensors allow the e-skin exhibit thinner thickness, simpler fabrication process, and greater stretchability (Table S1) [19, 48–52]. Experimental studies and numerical simulations of both electrical characteristics and mechanical properties of the e-skin prove the sensitive tactile sensing behaviors and excellent durability. The materials and devices presented in this work provide insights into the materials science of intrinsically stretchable functional materials, processing routes of stretchable devices and integration strategies of soft sensing arrays, and offer an efficient route for low-cost, large-area, high sensor density e-skin.

## 2. Result and Discussion

**Figure 1:**
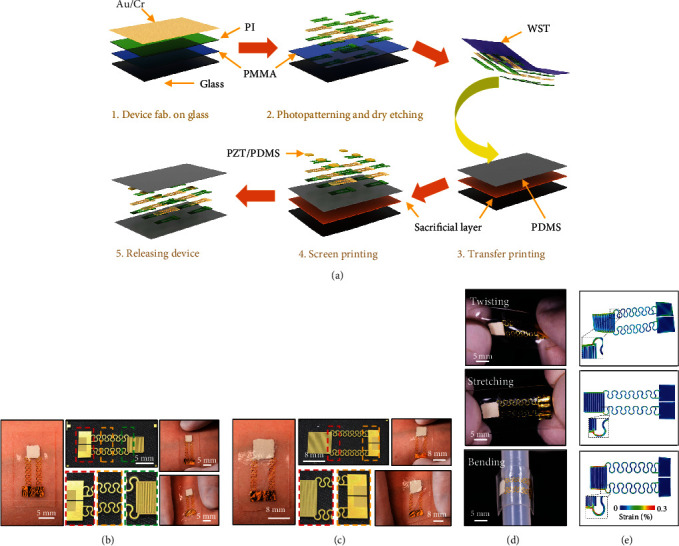
(a) Fabrication process flow of the intrinsically stretchable piezoelectric sensor for e-skin. (b, c) Photographs of two representative piezoelectric sensors with 5 × 5 mm^2^ (b) and 8 × 8 mm^2^ (c) sensing areas mounted onto a forearm; the enlarged images show the details of the electrode designs. (d, e) Images (d) and finite element modeling (e) of the device in bended, stretched, and twisted configurations.

Figure 1(a) presents the schematic illustrations of the fabrication process for the e-skin. The fabrication begins on a precleaned glass substrate, where a thin poly(methyl methacrylate) (PMMA, thickness, 200 nm) film spin-casted on the class serves as a sacrificial layer and a polyimide (PI; 2 *μ*m) layer formed by spin-casting supports the circuits and electronics on top. Then, in-plane gold/chromium (Au/Cr; 200 nm/40 nm) electrodes and structural designed interconnects developed by sputtering and photolithography on the PI supporting layer serve as electrodes and circuit interconnects. Another PI layer on top of the metallic traces patterned with selective area dry etching forms an encapsulation layer for the circuit. Dissolving the PMMA sacrificial layer enables lifting off the PI supported soft circuit and then transferring printed onto a soft, thin elastomer polydimethylsiloxane (PDMS) substrate. PZT powders blending with PDMS at a concentration of 85.8 wt.% serve as piezoelectric elastomer-sensing components (charge constant, 20 pC/N) and forms desired patterns on the flexible circuit via screen printing (Figure S1). The 85.8 wt.% PZT/PDMS composite has exhibited excellent mechanical characteristics (Figure S2). Another PDMS layer with the same thickness of the substrate coated on top encapsulates the electronics and greatly enhances the mechanical flexibility and durability [3]. The detailed fabrication process can be found in the Materials and Methods Section. The area of the intrinsically stretchable sensor is scalable according to different applications and different integration strategies. Figures 1(b) and 1(c) show the optical images of two representative single-unit sensors mounted on the forearm and the enlarged pictures of different working areas. The overall demission of these two sensors are 20 mm × 8.5 mm × 0.52 mm and 24.8 mm × 9.7 mm × 0.52 mm, where the tactile sensing areas are 25 mm^2^ and 64 mm^2^, respectively (Figure S3). The intrinsically stretchable nature of the sensors allows the e-skin to exhibit very robust mechanical properties that can undertake various deformations like human skin (Figure 1(b) and 1(c)). To further demonstrate its excellent mechanical compliance during deformation, the e-skin with a working area of 25 mm^2^ is subjected to typical deformations including twisting, stretching, and bending, as shown in Figure 1(d). Finite element analysis (FEA) guided the design of the serpentine interconnect layout where the thin Au/Cr layer is located in the neutral plane of the serpentine interconnects to decrease its strain level caused by the out-of-plane deformations, as shown in Figure 1(e). The elastic stretchability of the serpentine interconnects can achieves ~20%, i.e., the strain in the metal layer is less than its yield strains (0.3%) for 20% stretching. In the working area, the deformation is very small due to the PZT/PDMS composite being significantly stiffer (~1 MPa) than the PDMS encapsulation (~70 kPa) and the dense interdigitated electrodes. Further, the ends of the phantom skin are twisted by 100° and bent by 200° (~8 mm bending radius) in the FEA simulation to verify that the strain level in the metal layer does not exceed the yield strain, therefore highlighting the robust design of the device while operating under realistic physiological loads. Figure S4 shows the effects of surface roughness and thickness of the PZT/PDMS elastomer thin layer on the electrical performance of the 64 mm^2^ device, where we can find that either the smoother surface or thinner thickness yields greater voltage outputs.

**Figure 2:**
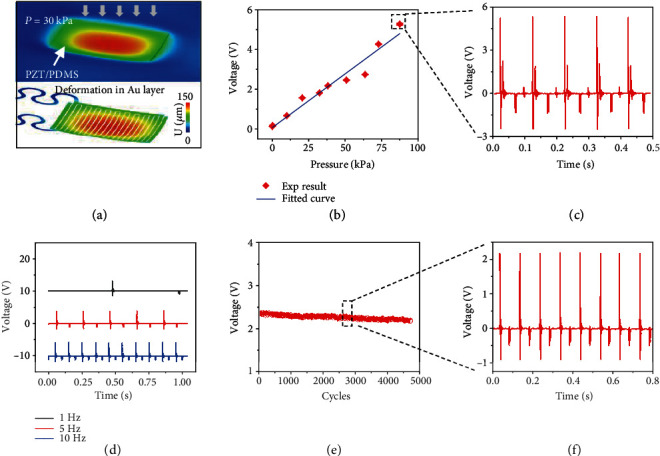
(a) The finite element analysis of a 5 × 5 mm^2^ device under a constant stress of 30 kPa onto its working area. (b) The electrical signal of the device versus applied stress at a constant frequency of 10 Hz. (c) Voltage output of the device as a function of time under stress of 87.5 kPa. (d) The electrical signal of the device versus time at a constant stress of 64 kPa under three different frequencies. (e) Peak values of each electrical output generated by the 64 mm^2^ device with over 4500 continuous working cycles under the load and frequency of 32.4 kPa and 10 Hz. (f) The details of the electrical signal in the marked region of (e).

Figure 2(a) shows the computered results of deformations in the sensor (top) and the Au layer (bottom) when pressure *P* = 30 *kPa* is applied to the working area. The maximum spacing among the electrodes increases by ~2.6% due to the stretched phantom skin induced by the applied pressure. Evaluation of the relationship between the voltage output of the sensor and the values of a set of stress that span those of tactile forces confirmed the linear response behavior of the senor to the external stress, as shown in Figure 2(b) and S5(a). Figure 2(b) and S5(a) present the peak voltage in 64 mm^2^ and 25 mm^2^ sensors as a function of various applied touched forces from 0.1 kPa to 110.4 kPa. The sensitivity of the sensors can be calculated from the fitted curve as 0.067 V/kPa for 64 mm^2^ devices and 0.01 V/kPa for 25 mm^2^ devices, respectively. Figure 2(c) shows the voltage versus time of the sensor with the applied stress of 87.5 kPa, where the device yields a peak voltage output of 5.2 V with great signal-to-noise ratio, indicating its high sensitivity and effective responsivity to external stimuli (Figure 2(c)). Figure 2(d), S6 and S7 show the electrical signal versus time with three different frequencies (1 Hz, 5 Hz, and 10 Hz) at a constant stress of 64 kPa. It is interesting that the voltage output increases with frequency, because the same load amplitude increases the amount of work performed by the external force [53]. Similar to the tendency observed in Figure S6, the open-circuit voltage output yielded by the 25 mm^2^ device reaches to the highest value as the frequency approaches to 10 Hz at a constant stress of 110.4 kPa, as shown in Figure S5(b). By analyzing its electrical response at a constant stress of 87.5 kPa with three different frequencies, the response time ranges from 0.45 ms to 0.72 ms, as shown in Figure S8. Figure 2(e) shows the output voltage peak of the device under multiple cycles (>4500) of pressing at 32.4 kPa and 10 Hz, where the voltage amplitude after thousands of cycles of pressing still maintain its initial values, proving the great stability and durability. Figure 2(f) shows the details of the electrical signal marked in Figure 2(e).

**Figure 3:**
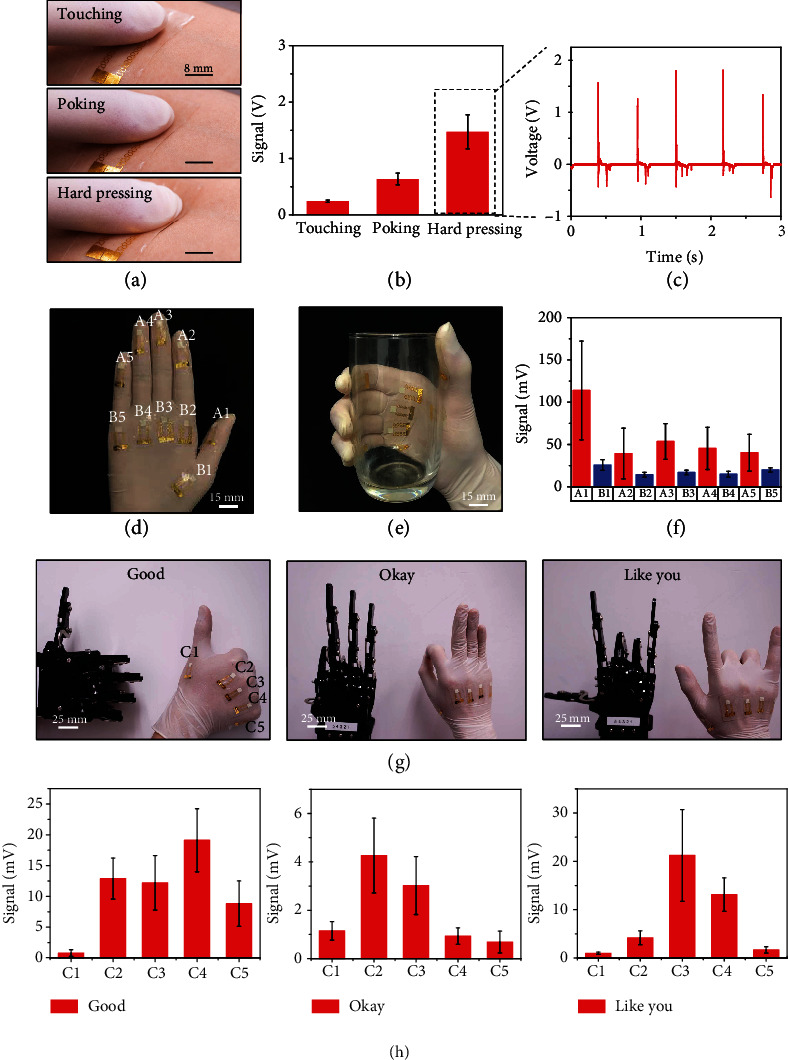
(a) Optical images illustrating three different external loads of touching (~0.5 kPa), poking (~13.4 kPa), and hard pressing (~27.2 kPa) on a piezoelectric elastomer sensor. (b, c) Electrical responses in the piezoelectric elastomer sensors to the three kinds of touching loads. (d) Optical image of a glove with 10 sensors mounted on different locations. (e) Photo of the sensors mounted on the glove grabbing a glass. (f) Electrical signals outputs of the 10 devices mounted on the glove when holding the glass. (g) Optical images of three gestures with 5 devices attached to the glove. (h) Voltage outputs of the 5 sensors mounted on the glove under gestures of “Good”, “Okay,” and “Like you”.

Demonstration of e-skin testing involved in mounting the single-sensor device (64 mm^2^) on a volunteer's forearm and measuring the voltage responses to three types of typical tactile forces including touching, tapping, and hard pressing by a finger are shown in Figure 3(a). Due to the thin and soft features of the e-skin sensor, conformal contact without delamination from the skin was maintained during the entire measuring cycles of touching, tapping and hard pressing (Figure S9). Analysis of voltage outputs from the e-skin sensor stimulated by touching, tapping, and hard pressing is shown in Figure 3(b), where the device can accurately distinguish various tactile forces, with the pressure detection as low as 0.5 kPa (gentle touching). Intense force, i.e., finger hard pressing (10 kPa to 27.2 kPa) at a constant frequency of 2 Hz yields voltage outputs ranging from 0.63 V to 1.47 V, providing the sensor's self-powering capabilities (Figure 3(c)). To further investigate its electrical characteristics, a constant external trigger (hard finger pressing) was applied onto the device at four different stretching levels, including 0%, 2.8%, 8.3%, and 16.7%, as shown in Figure S10, and it is found that stretching the device has a negligible effect on its pressure sensing output. As shown in Figure S11, the uniform signal outputs, stimulated by hard finger pressing at three different locations of the 64 mm^2^ device, demonstrate its electrical anisotropy at the working area. Next, 10 small singe-unit sensors devices (working area, 25 mm^2^) were integrated onto different locations of latex gloves through van der Waals forces by a simple transfer method, which can provide information of voltage outputs for analyzing force distributions while grabbing an objective (Figures 3(d) and 3(e)). As shown in Figure 3(f), the voltage outputs of 10 sensors range from 14.1 mV to 113.8 mV when grabbing an empty cup. The sensor on the thumb (A1) yields the highest voltage output of ~113.8 mV on average and other sensors yield smaller voltage outputs of tens of mV. Such result clearly reveals the force distribution thereby proving the excellent performance and potentials for e-skin. Using the voltage outputs generated from finger motions, this enables the e-skin to act as an interface for human-machine integration. Various gestures of a robotic hand (uHand2.0, Shenzhen Hiwonder Co., Ltd.) with precise amplitudes and motions can be duplicated with a human hand while wearing the sensors in the latex gloves. As shown in Figures 3(g) and S12, the sensing voltage generated by the five individual sensors C1 to C5 are used to control the corresponding robotic fingers, including the thumb, forefinger, middle finger, ring finger, and little finger, through the Arduino board. Figure 3(g) shows a set of gestures, representing “Good,” “Okay,” and “Like you,” that translate to the hand by specific finger gestures and the corresponding voltage signals of these sensors are shown in Figure 3(h). With real-time data communication and algorithmic analysis, the robotic hand can identify and imitate the gestures of the human hand without time delay (Movie. S1).

**Figure 4:**
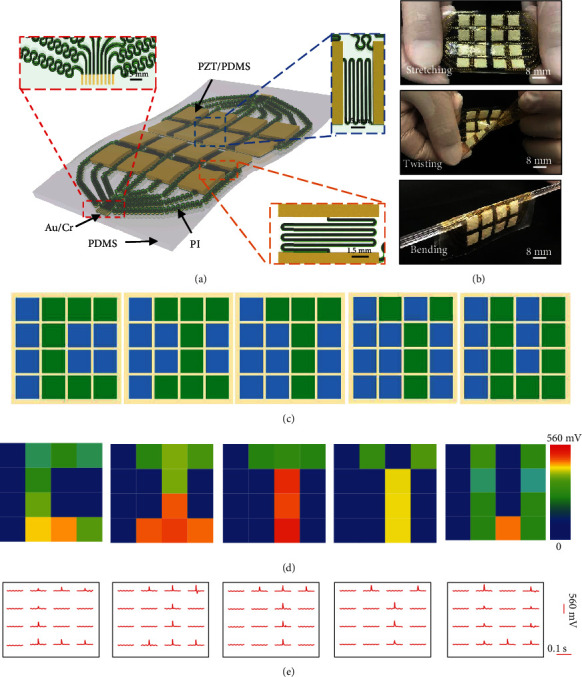
(a) Schematic illustration of an e-skin device with a 4 × 4 tactile sensor array. (b) Optical images of the device under three mechanical deformations, including stretching, twisting, and bending. (c–e) Characterizations of the e-skin device under a touching pattern of “CITYU” (c) and the voltage output distributions of 16 sensors (d, e).

The first demonstration of the e-skin sensing array was investigated by using a stretchable sheet (70 mm × 45 mm) with 16 sensors arranged at 4 × 4, where the working area of each sensor is 64 mm^2^ (Figure 4(a)). This e-skin sensing array adopted the same device layout as the single-unit sensor (Figure S13). The ultrathin, soft PDMS substrate of the e-skin allows the device to conformally laminate onto the human skin through van der Waals forces without any irritation (Figure S14). The intrinsically stretchable nature of the piezoelectric sensors and advanced serpentine design of the metallic interconnects offer great flexibility and stretchability for skin integration. The device can still survive and maintain normal functions even after extreme mechanical force loadings, such as continuous stretching (~8%), twisting (~90°), and bending (180° at a radius of 3 mm) (Figure 4(b)). Figure S15 presents the electrical outputs of a 4 × 4 sensor array under the original state, bending for 100 and 200 cycles, and twisting for 100 and 200 cycles at a constant pressure and frequency of 10 kPa and 4 Hz. The unchanged signal outputs further prove the stability of the 4 × 4 sensor array. Figures 4(c)–4(e)show voltage responses in the sensing array for a representative touching pattern “CITYU” by a finger. It is clear that the e-skin is very sensitive to finger touching with obvious voltage output, affording the skin-like platform with capabilities to rebuild the tactile information.

**Figure 5:**
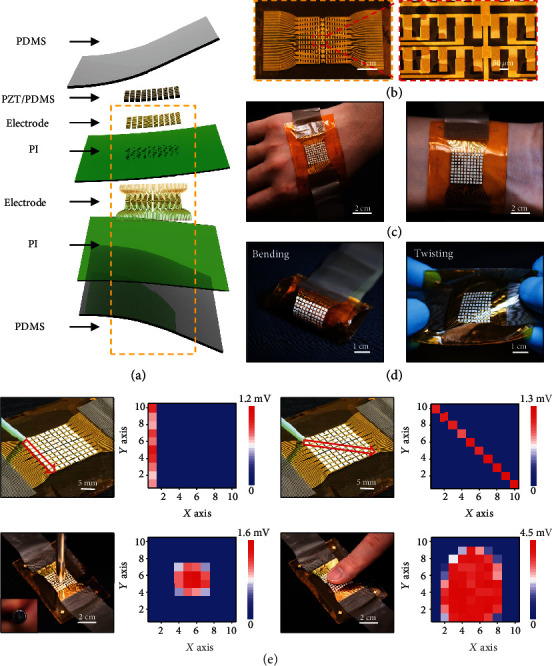
(a) Schematic illustration of the explosive view of an e-skin with a 10 × 10 tactile sensor array. (b) Optical images of the e-skin device and the enlarged photo of the electrode area. (c) Optical images of the device mounted onto the human skin. (d) Optical images of the device under two mechanical deformations of twisting and bending. (e) Voltage output distributions of the 10 × 10 sensors touched by different objects and different ways, including a plastic rod sliding along straight line and diagonal, a metal bar pressing, and finger touching.

Multiplexed device designs allow high-resolution and high-channel count e-skin interfaces by simplifying device geometry for tactile information acquisition (self-developed software, Figure S16). Here, the piezoelectric elastomer not only provides a great choice for sensors but also offers a scalable pathway towards large-area fabrication. A high sensing resolution embodiment with 100 channel sensors in a 2 cm × 2 cm area appears in Figure 5(a). This embodiment includes a 10 × 10 sensor array, contributing to a high density of 25 devices per square centimeter. A slightly difference from the layout of the aforementioned 16-channel device, an additional thin PI layer separates the conducting electrode and the interconnects for more compact integration. Selective area dry etching of this PI layer forms holes for connecting in-plane electrodes of the piezoelectric sensors and acquiring sensing data (Figure 5(b), S17). It is worth to mention the lot and functional piezoelectric sensor yields are both 100%, respectively, which can be attributed to the simple processing route used in this work. Figure 5(c) presents the optical images of a device mounted on the back hand and wrist of an examiner. More than a thousand bending and twisting cycles of the device did not affect the sensing performance, showing its robust mechanical properties (Figure 5(d)). Figure S18 summarizes the electrical signal outputs in the 10 × 10 sensor array tested under five mechanical deformations, including original state, bending for 100 and 200 cycles, and twisting for 100 and 200 cycles, at a constant pressure and frequency of 92.5 kPa and 4 Hz. Figure S19 presents the peak voltage by a unit of the 10 × 10 sensor array as a function of various applied touched forces from 12.9 kPa to 120 kPa with a sensitivity of 0.00018 V/kPa. The low sensitivity of the unit may mainly result from the middle PI layer, greatly impeding the strain level of the working area. Figure S20 shows the sensitivity of each unit of the 10 × 10 sensor array, ranging from 0.00016 V/kPa to 0.00019 V/kPa. Figure 5(e) summarizes the electrical responses of the device to various external triggers, including sliding in vertical and diagonal ways by a plastic rod (12.9 kPa), pressing by a metal bar (17.3 kPa), and a fingertip (~27.2 kPa). The spatial distribution of the amplitude of the evoked voltages measured by the e-skin sensing array is consistent with the tactile information, including the contact area and shape of the pressing objects as described with the color map (Figure 5(e)). Benefited from this platform, e-skin with the same materials and device architectures was also adapted to realize a larger area (4.2 cm × 4 cm) and 256 channel counts (16 × 16 sensor array) with a potential for applications that required large tactile sensing (Figure S21). To fabricate higher density of sensor arrays, decreasing the electrode width with resolution as high as several micrometer can be considered in the future.

## 3. Conclusion

In summary, we introduce a concept of increased channel counts e-skin with simple processing that build a foundation of the capabilities in skin-integrated device for accurate and high-resolution tactile sensing demonstrated by multiple hand motions/deformations and pressure sensing scenarios. By combining the advances in materials engineering, mechanic design and device integration, a high-throughput sensor with a large area and scalable e-skin was realized. The use of intrinsically stretchable piezoelectric elastomer sensors aligns with flexible electronics technology and offers potential applications in wearable technologies, health monitoring, and human-machine interfaces.

## 4. Materials and Methods

### 4.1. Fabrication of Piezoelectric Elastomer Composite

The piezoelectric elastomer sensors consist of PZT powders and PDMS, where the PZT powder (diameter of 0.9 *μ*m on average) and PDMS (Sylgard 184) were purchased from Xi'an Yisheng electronics Co. Ltd., China, and Dow Corning Corporation. PZT (10 g), and PDMS (1.5 g, crosslink ratio of 10 : 1) was poured into a speed mixer and stirred at a speed of 500 rpm for 1 h to form elastomer precursors. Then, this mixture was transferred into an agate mortar and subsequently grinded for 1 h at room temperature. After full dispersion, the PZT/PDMS composite was poured into marked beakers for film casting.

### 4.2. Assembly of the Single and 4 × 4 Array Devices

The fabrication started on a quartz glass, which was first cleaned by acetone, alcohol, and deionized water (DI water) sequentially. A PMMA thin film was spin coated onto the glass at 2000 rpm (20 mg/ml) for 30 s and then baked on a hotplate at 200°C for 20 min, which served as the sacrificial layer. A thin layer of PI was spin coated on the PMMA (poly, amic acid solution 12.0 wt. % ±0.5 wt. %, 2 *μ*m) at 3000 rpm for 30 s, baked at 250°C for 30 min. Next, Au/Cr (thickness, 200/40 nm; width, 50 *μ*m) was sputtered onto the PI film and then patterned by photolithography and etching, yielding metal traces in the desired geometries. Here, a positive photoresist (PR, AZ 5214, AZ Electronic Materials) was spin coated at 3000 rpm for 30 s, soft bake on a hot plate at 110°C for 4 min, then exposed to ultraviolet light for 5 s, and finally developed for 15 s in a developer (AZ 300MIF). After developing, the PR was removed by acetone and rinsed with DI water. Then, we spin casted another layer of PI (2 *μ*m, 3000 rpm for 30 s, annealed at 250°C for 30 min) and then selectively etched by Oxford Plasma-Therm 790 RIE system (patterns defined by photolithography similar as previous step) at the power of 200 W for 10 min, forming encapsulation layers for all interconnect areas besides the electrodes areas. Immersing the sample in acetone for 12 h dissolved the PMMA layer. Next, water-soluble tapes (WSTs) were used to pick up the patterns. We exposed the receiving PDMS substrates (PDMS: crosslink =30 : 1) and the WSTs to UV-induced ozone to create chemical groups between the electrodes and PDMS substrates to enhance the bonding strength. Attaching the WSTs on the PDMS and then heated in an oven at 70°C for 10 min formed strong bonding. Immersing the sample in water to remove the WSTs and realized soft stretchable electrodes. Next, piezoelectric elastomer precursors were screen-printed onto the stretchable electrodes via screen printing through a laser cut steel mask forming 0.3 mm thick sensors. After scree -printing the piezoelectric elastomer precursor, the sample was heated at 120°C for 30 min until the PZT elastomer was completely cured. Finally, the top PDMS (PDMS: crosslink =30 : 1) encapsulation layer with a thickness of 0.11 mm was spin coated and cured.

### 4.3. Assembly of the High-Channel Count Device

The fabrication started on a precleaned quartz glass. We attach a thin film of PI (thickness, 18 *μ*m) on the glass supporting the substrate by a double-sided tape, then cleaned by ethanol. Next, Au/Cr (thickness, 200/40 nm; width, 500 *μ*m) was sputtered onto the PI film and then patterned by photolithography and etching, yielding metal traces in the desired geometries (the same process and parameters as mentioned above). Then, we spin casted another layer of PI (2 *μ*m) and then selective dry etching formed an encapsulation layer with small holes for connecting electrodes. We sputter another layer of Au/Cr (200 nm/40 nm) on the PI layer and then patterned by photolithography and etching, yielding an in-plane electrode. After releasing the sample from the glass sheet directly, we attach the sample onto a PDMS substrate. Next, the piezoelectric elastomer precursor was screen printed onto the electrodes via screen printing through a laser cut steel mask (thickness, 0.3 mm), followed by 120°C heating for 30 min until the piezoelectric elastomer was completely cured. Finally, the top PDMS encapsulation layer with a thickness of 0.11 mm was spin coated and cured.

### 4.4. Characterization

The piezoelectric charge constant (*d*_33_) of the PZT/PDMS elastomer was measured by YE2730A *d*_33_ meter. The voltage outputs of the single and 4 × 4 array devices were collected by a PL3516/P Powerlab 16/35 with a constant sampling frequency of 2 × 10^4^ Hz. The voltage output data of the high-channel count devices was collected by a NI PCI-6255 Data Acquisition Card. The elastic modulus of the PZT/PDMS composite was measured using a material testing machine (Lloyd LS1, AMETEK, USA) with an elongation speed of 50 mm/min at room temperature. The dimension of the composite is 50 × 10 mm. The external pressure was measured by a force sensor (NTJL-1, Nanjing Tianguang Electric Technology Co. Ltd.). The pressure by touching, tapping, and hard pressing are 0.5 kPa, 10 kPa, and 27.2 kPa, respectively.

### 4.5. Mechanical Simulations of the Stretchable Device

Finite element analysis (FEA) was implemented in ABAQUS (Analysis User's Manual 2016) to optimize the design layout to decrease the strain level in the Au/Cr layer for stretching, twisting, and bending skin deformations. The PDMS, PZT/PDMS composite, and phantom skin were modelled by hexahedron elements (C3D8R) while the thin Au/Cr layer (240 nm thick) and PI layers (2 *μ*m) were modelled by shell elements (S4R). The minimal element size was 1/4 of the width of the Au/Gr wires (200 nm), which ensured the convergence and the accuracy of the simulation results. The *d*_33_ of the PZT/PDMS elastomer used in the analysis is 25 pC/N (measured *d*_33_ meter). The elastic modulus (*E*) and Poisson's ratio (*ν*) used in the analysis were *E*_PDMS_ = 70 kPa, *ν*_PDMS_ = 0.5, *E*_PI_ = 2.5 GPa, *ν*_PI_ = 0.34, *E*_Au_ = 79 GPa, *ν*_Au_ = 0.4, *E*_Cr_ = 270 GPa, *ν*_Cr_ = 0.21, *E*_PZT/PDMS_ = 1 MPa, *ν*_PZT/PDMS_ = 0.46, *E*_Skin_ = 130 kPa, and *ν*_Skin_ = 0.5.

## Supplementary Materials

Supplementary 1 Table S1: the summary of flexible piezoelectric pressure sensor based on sandwich and in-plane electrode design. Figure S1: the schematic diagram of the screen printing technology. Figure S2: the exploded-view schematic illustration of the single device with 25 mm^2^ working area. Figure S3: the electrical signals of the device (25 mm^2^ working area) versus (a) stress at a constant frequency of 10 Hz and (b) frequency at a constant stress of 110.4 kPa. Figure S4: the electrical signals of the 64 mm^2^ device versus frequency at a constant stress of 64 kPa. Figure S5: the electrical response of the 64 mm^2^ device under the constant stress of 64 kPa with three different frequencies. Figure S6: the enlarged electrical response of the 64 mm^2^ device at the constant stress of 87.5 kPa with three different frequencies. Figure S7: the optical images of the 64 mm^2^ device mounted onto the human skin under finger hitting. Figure S8: the electrical signal of the 64 mm^2^ device mounted onto the human skin with four different stretching levels under a constant stress and frequency of 27.2 kPa and 2 Hz. Figure S9: the electrical response of the 64 mm^2^ device mounted onto the human skin under a constant stress and frequency of 27.2 kPa and 2 Hz with the three different locations. Figure S10: (a) the schematic diagram of the testing circuit for controlling robotic hand. (b) The self-developed software interface for controlling the robotic hand. Figure S11: the exploded-view schematic diagram of the 4 × 4 array device. Figure S12: the optical images of the 4 × 4 array device mounted onto the forearm of an examiner. Figure S13: (a) the schematic diagram of the testing circuit for measuring the electrical signal of the multiplexed device. (b) The presence of the self-developed software interface as a finger is tapping on the multiplexed device. Figure S14: the enlarged optical image of the connection area between the 10 × 10 array device and ACF cables. Figure S15: the optical images of the 1616 array device.

Supplementary 2 Movie S1: Robotic hand controlling performed by the flexible e-skin.
