# Supplementary material for: Electronic Skin from High-Throughput Fabrication of Intrinsically Stretchable Lead Zirconate Titanate Elastomer
Source: Research (Wash D C). 2020 Oct 17;2020:1085417. doi: 10.34133/2020/1085417 (PMC7586250; doi:10.34133/2020/1085417)
Supplement: Supplementary 1 — Table S1: the summary of flexible piezoelectric pressure sensor based on sandwich and in-plane electrode design. Figure S1: the schematic diagram of the screen printing technology. Figure S2: the exploded-view schematic illustration of the single device with 25 mm2 working area. Figure S3: the electrical signals of the device (25 mm2 working area) versus (a) stress at a constant frequency of 10 Hz and (b) frequency at a constant stress of 110.4 kPa. Figure S4: the electrical signals of the 64 mm2 device versus frequency at a constant stress of 64 kPa. Figure S5: the electrical response of the 64 mm2 device under the constant stress of 64 kPa with three different frequencies. Figure S6: the enlarged electrical response of the 64 mm2 device at the constant stress of 87.5 kPa with three different frequencies. Figure S7: the optical images of the 64 mm2 device mounted onto the human skin under finger hitting. Figure S8: the electrical signal of the 64 mm2 device mounted onto the human skin with four different stretching levels under a constant stress and frequency of 27.2 kPa and 2 Hz. Figure S9: the electrical response of the 64 mm2 device mounted onto the human skin under a constant stress and frequency of 27.2 kPa and 2 Hz with the three different locations. Figure S10: (a) the schematic diagram of the testing circuit for controlling robotic hand. (b) The self-developed software interface for controlling the robotic hand. Figure S11: the exploded-view schematic diagram of the 4 × 4 array device. Figure S12: the optical images of the 4 × 4 array device mounted onto the forearm of an examiner. Figure S13: (a) the schematic diagram of the testing circuit for measuring the electrical signal of the multiplexed device. (b) The presence of the self-developed software interface as a finger is tapping on the multiplexed device. Figure S14: the enlarged optical image of the connection area between the 10 × 10 array device and ACF cables. Figure S15: the optical images of the 1 [file 1085417.f1.docx]

Supplementary Materials

Electronic Skin from High-Throughput Fabrication of Intrinsically Stretchable Lead Zirconate Titanate Elastomer

Yiming Liu^1^, Huanxi Zheng^2^, Ling Zhao^1^, Shiyuan Liu^2^, Kuanming Yao^1^, Dengfeng Li^1^, Chunki Yiu^1^, Shenghan Gao^3^, Raudel Avila^4^, Chirarattananon Pakpong^1^, Lingqian Chang*^5^, Zuankai Wang*^2^, Xian Huang*^3^, Zhaoqian Xie*^6^, Zhengbao Yang*^2^, and Xinge Yu*^1^

1 Department of Biomedical Engineering

City University of Hong Kong

Hong Kong 999077, People’s Republic of China

2 Department of Mechanical Engineering

City University of Hong Kong

Hong Kong 999077, People’s Republic of China

3 Department of Biomedical Engineering,

Tianjin University

Tianjin 300000, People’s Republic of China

4 Department of Mechanical Engineering

McCormick School of Engineering

Northwestern University

Evanston, IL 60208, USA.

5 School of Biology Science and Medical Engineering,

Beihang University

Beijing, 100191, People’s Republic of China

6 State Key Laboratory of Structural Analysis for Industrial Equipment,

Department of Engineering Mechanics,

International Research Center for Computational Mechanics,

Dalian University of Technology,

Dalian 116024, People’s Republic of China

E-mail: zuanwang@cityu.edu.hk (ZW), huangxian@tju.edu.cn (XH) zxie@dlut.edu.cn (ZX), [zb.yang@cityu.edu.hk](mailto:zb.yang@cityu.edu.hk) (ZY) and [xingeyu@cityu.edu.hk](mailto:xingeyu@cityu.edu.hk) (XY)

**Key words:** electronic skin, screen printing, intrinsically stretchable, PZT elastomer

**Table. S1.** The summary of flexible piezoelectric pressure sensor based on sandwich and in-plane electrode design.

| Electrode Design | Functional Material | Pressure limit | Maximum  Stretchability | Reference |
| --- | --- | --- | --- | --- |
| Sandwich | PZT nanofibers | 0 ~ 100 kPa | unstretchable | [48] |
| Sandwich | Glycine-Chitosan | 5 ~ 60 kPa | unstretchable | [49] |
| Sandwich | SEBSm-PEI-PZT | 1 ~ 30 kPa | unstretchable | [50] |
| Sandwich | BaTiO_3_/PVDF | ≤33 kPa | unstretchable | [51] |
| Sandwich | III-N thin film | N.A. | unstretchable | [52] |
| In-plane | PZT/PDMS/Graphene | 2.5 ~ 150 kPa | Up to 15.2% | [19] |
| In-plane | PZT/PDMS | 0.4 ~ 110.4 kPa | Up to 20% | 25 mm^2^ device |


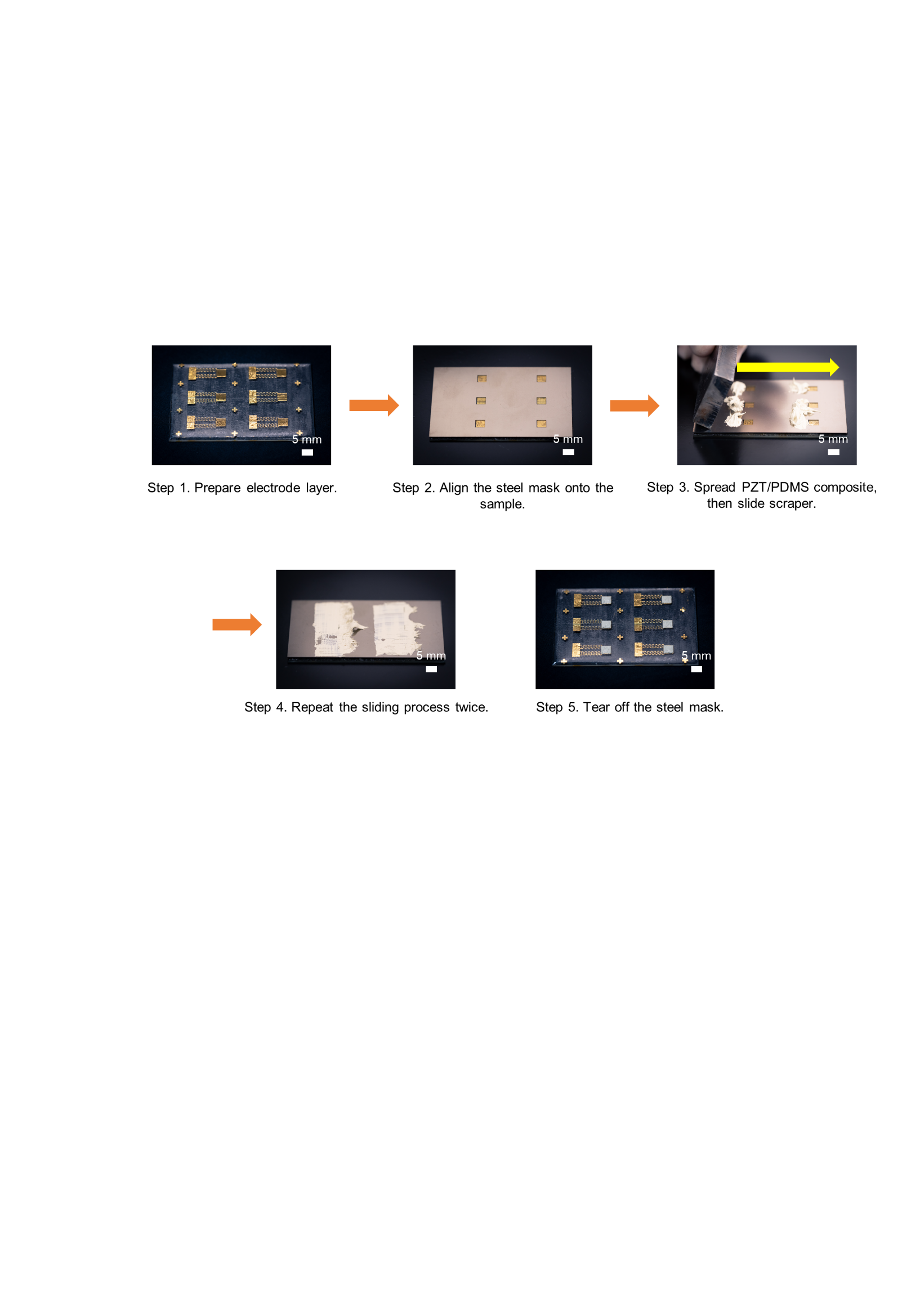


**FIGURE S1.** The schematic diagram of the screen-printing technology.


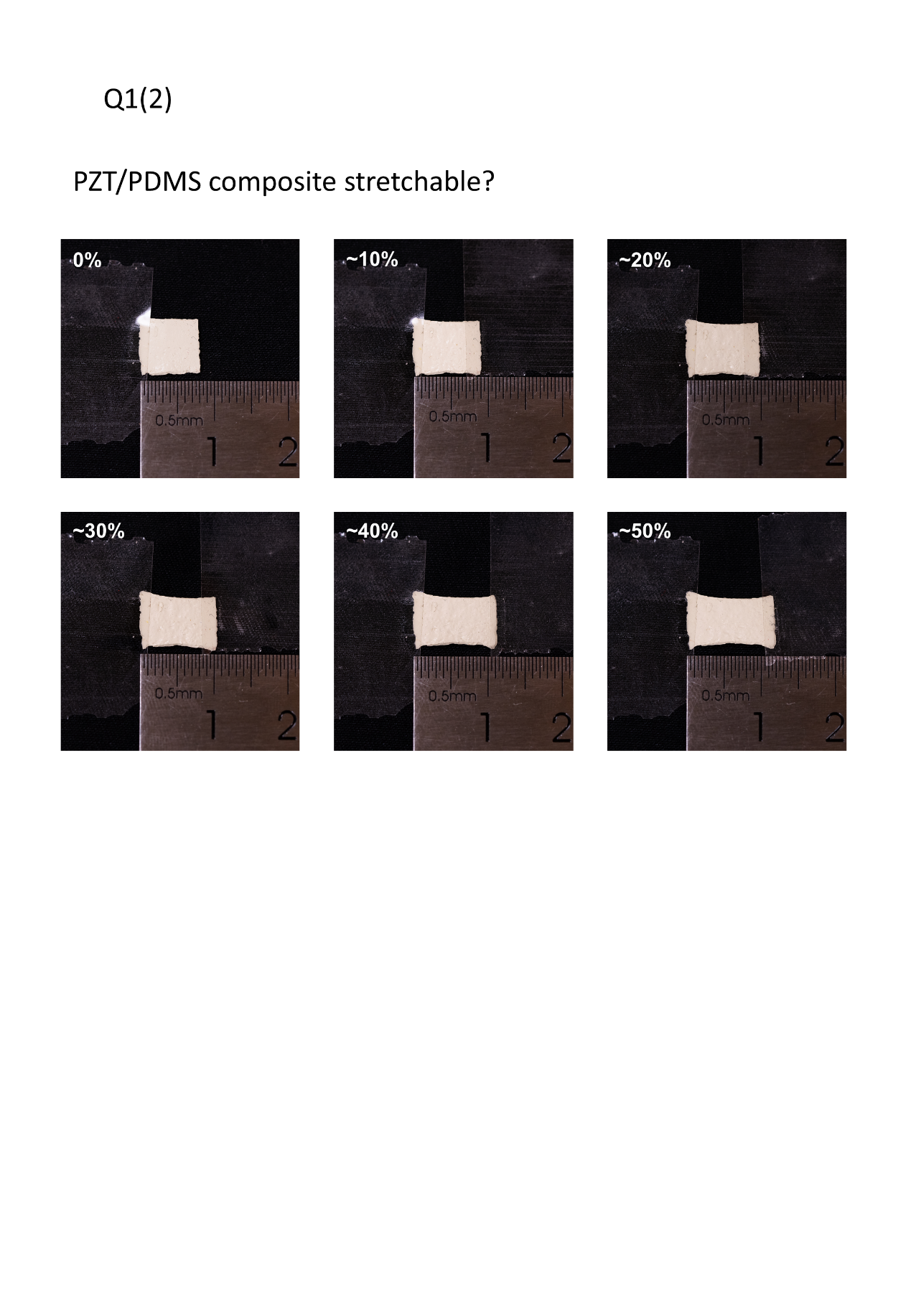


**FIGURE S2.** The optical images of the 85.8 wt.% PZT/PDMS composite under six stretchable states, including stretching 0, 10%, 20%, 30%, 40%, 50%, and 60%.


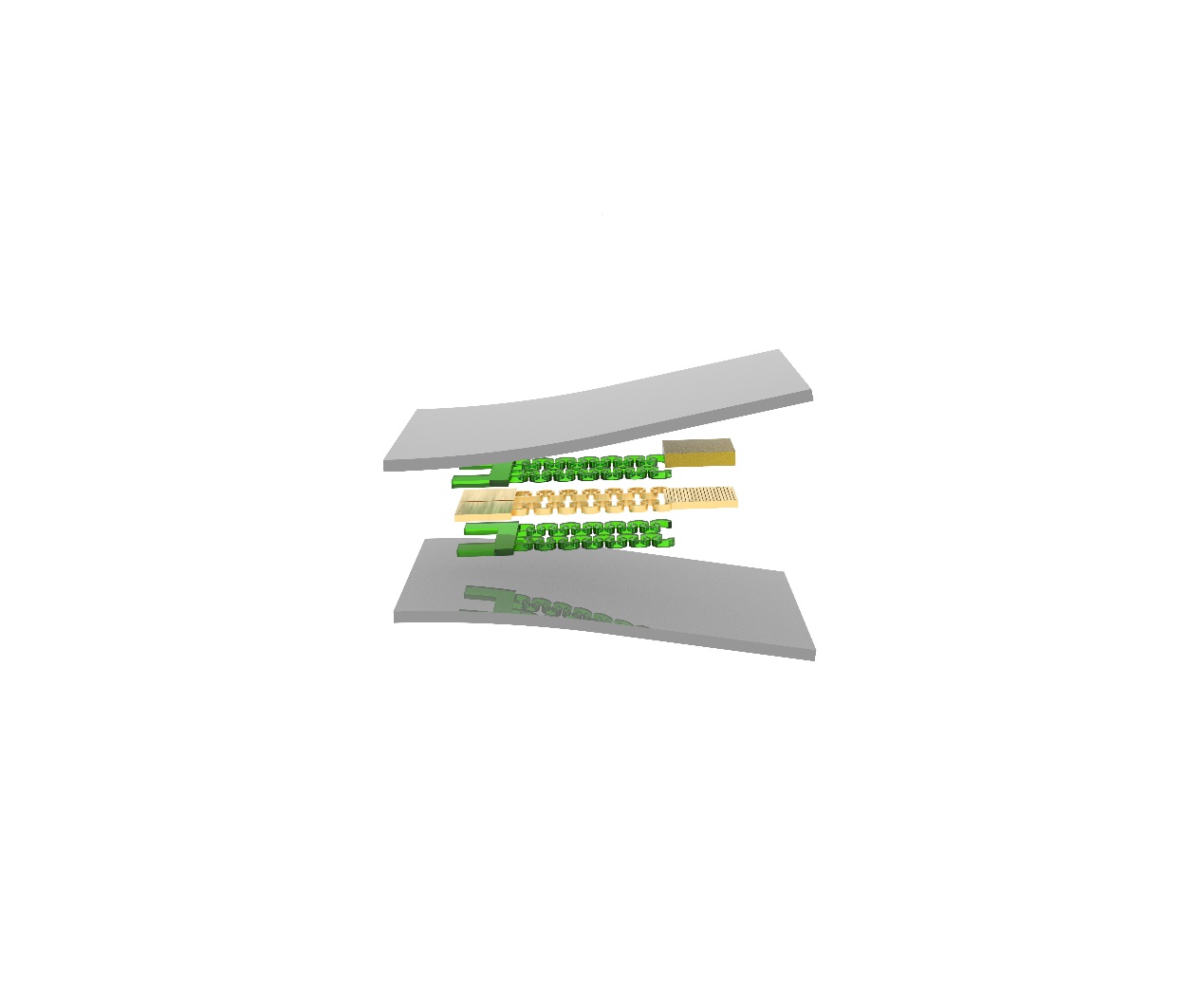


**FIGURE S3.** The exploded-view schematic illustration of the single device with 25 mm^2^ working area.


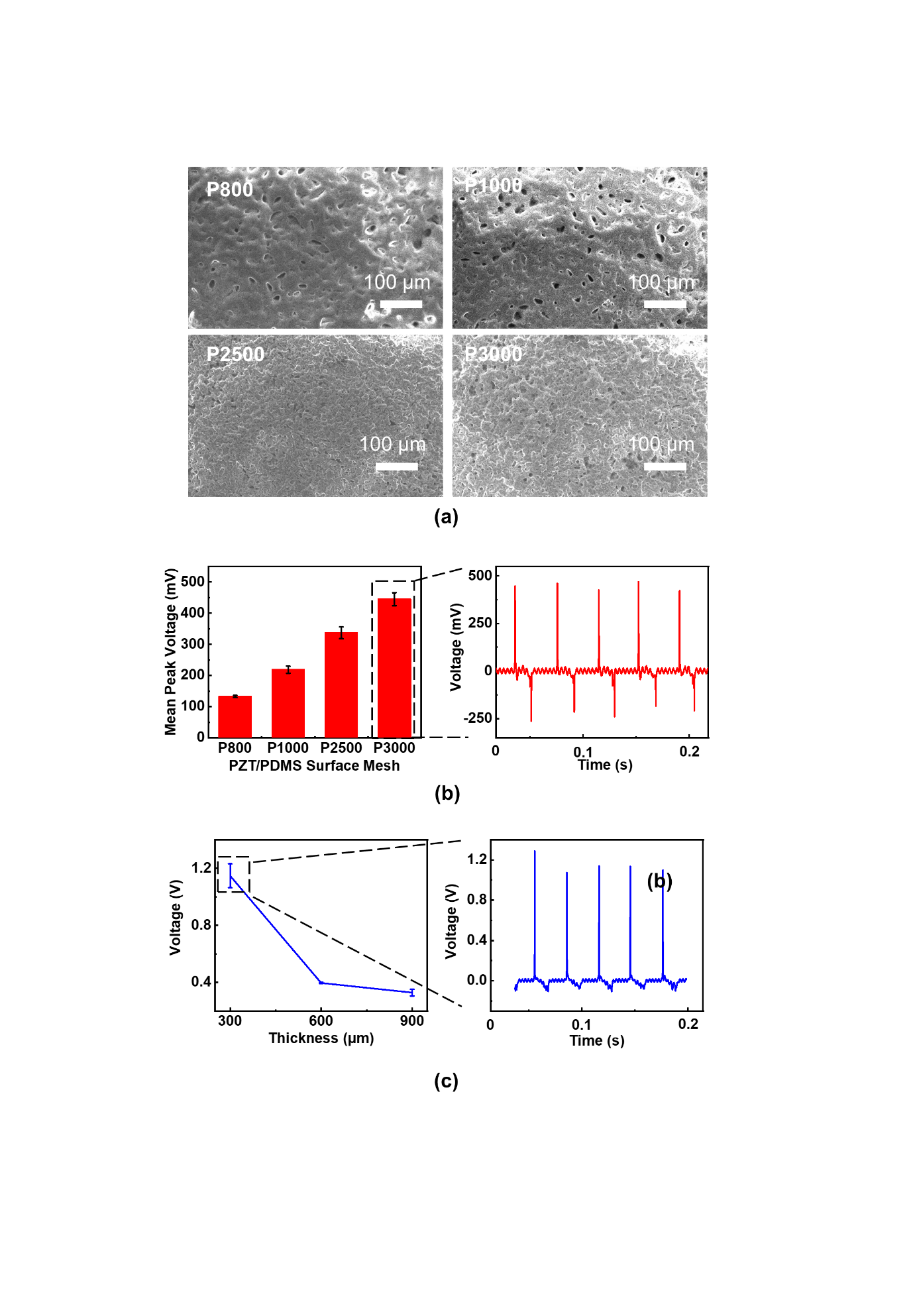


**FIGURE S4.** a) The SEM images of the four PZT/PDMS elastomer with different surface roughness. b) The electrical signals of the devices incorporating the four PZT/PDMS composite with different surface roughness, shown in a, at a constant pressure and frequency of 7 kPa and 10 Hz c) The electrical signals of the device with the three PZT/PDMS elastomer thickness, including 0.3 mm, 0.6 mm, and 0.9 mm at a constant pressure and frequency of 15 kPa and 10 Hz.


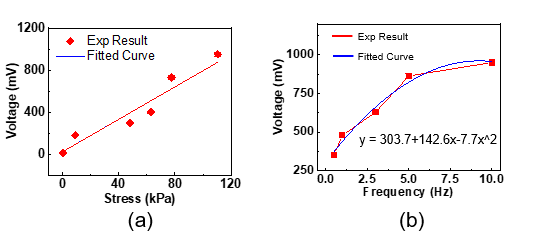


**FIGURE S5.** The electrical signals of the device (25 mm^2^ working area) verse (**a**) stress at a constant frequency of 10 Hz, (**b**) frequency at a constant stress of 110.4 kPa.


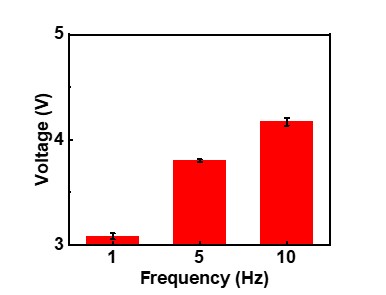


**FIGURE S6.** The electrical signals of the 64 mm^2^ device verse frequency at a constant stress of 64 kPa.


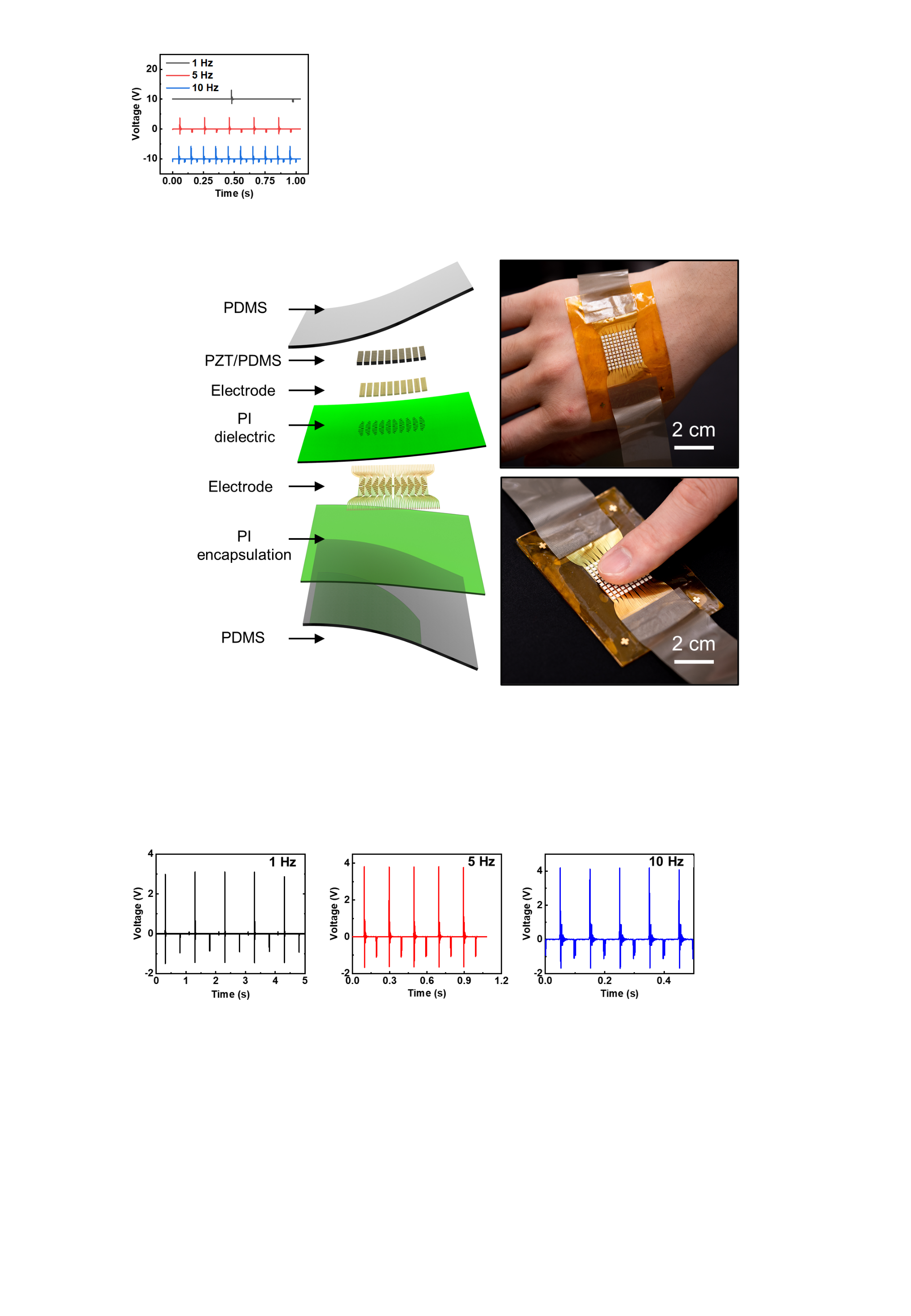


**FIGURE S7.** The electrical response of the 64 mm^2^ device under the constant stress of 64 kPa with three different frequencies.


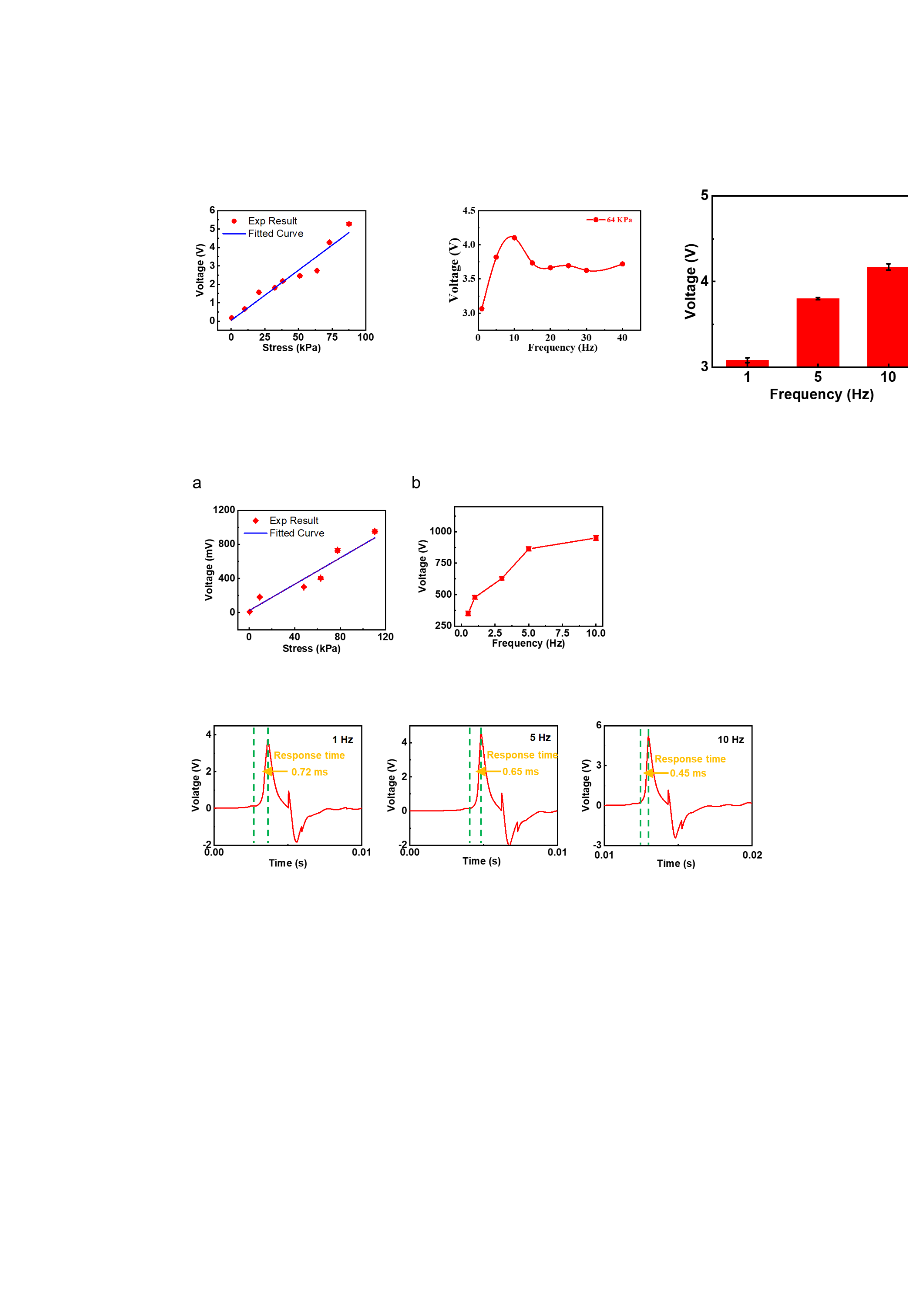


**FIGURE S8.** The enlarged electrical response of the 64 mm^2^ device at the constant stress of 87.5 kPa with three different frequencies.


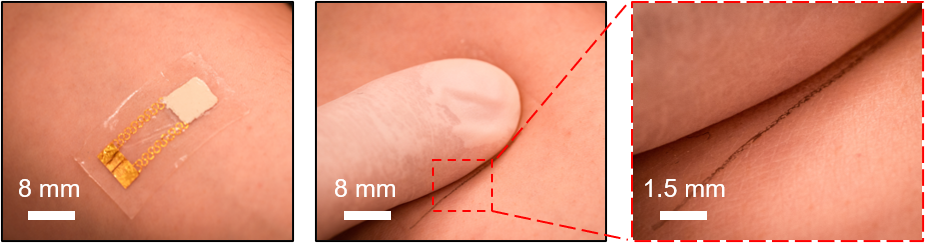


**FIGURE S9.** The optical images of the 64 mm2 device mounted onto the human skin under finger hitting.


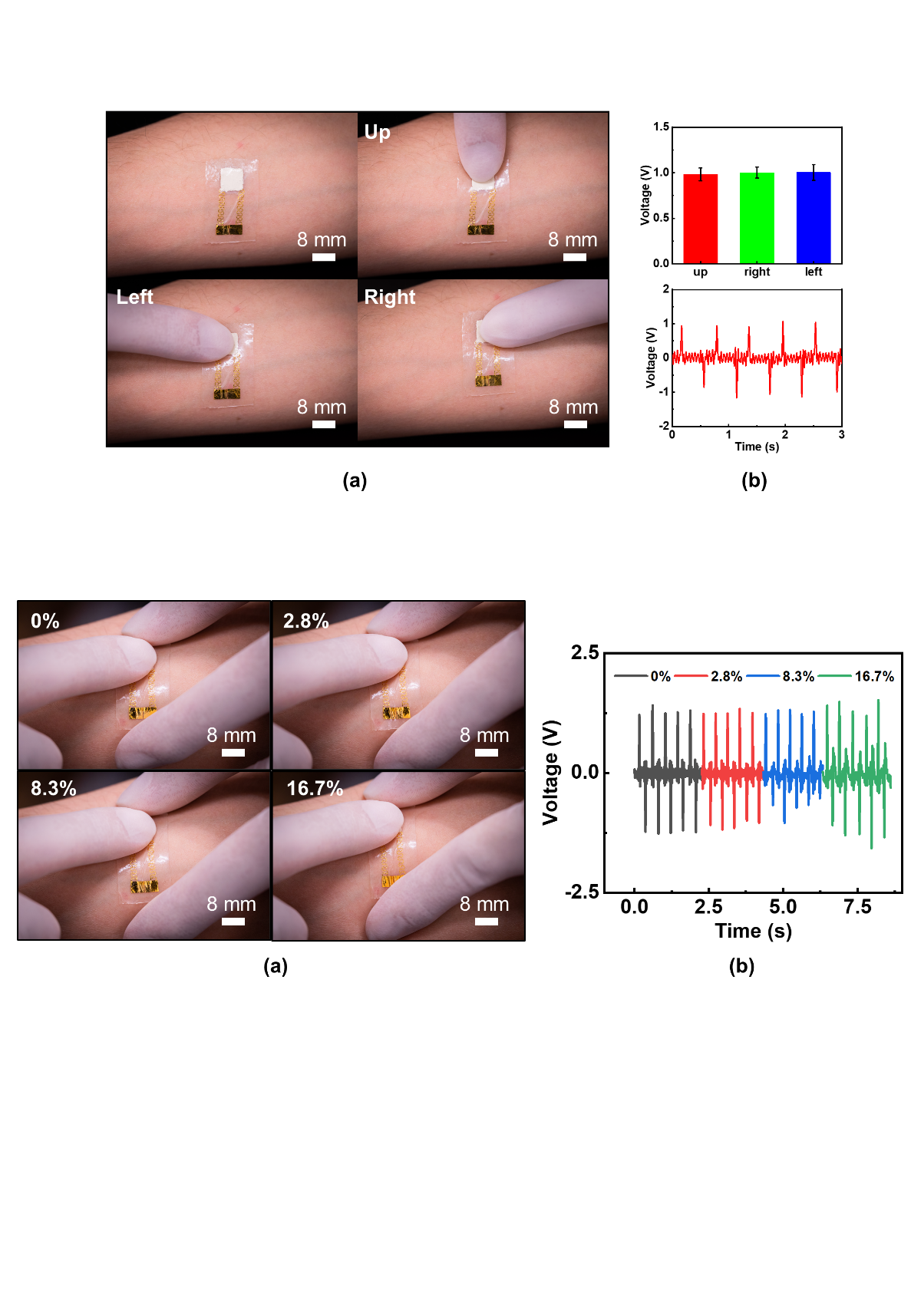


**FIGURE S10.** The electrical signal of the 64 mm^2^ device mounted onto the human skin with four different stretching levels under a constant stress and frequency of 27.2 kPa and 2 Hz.


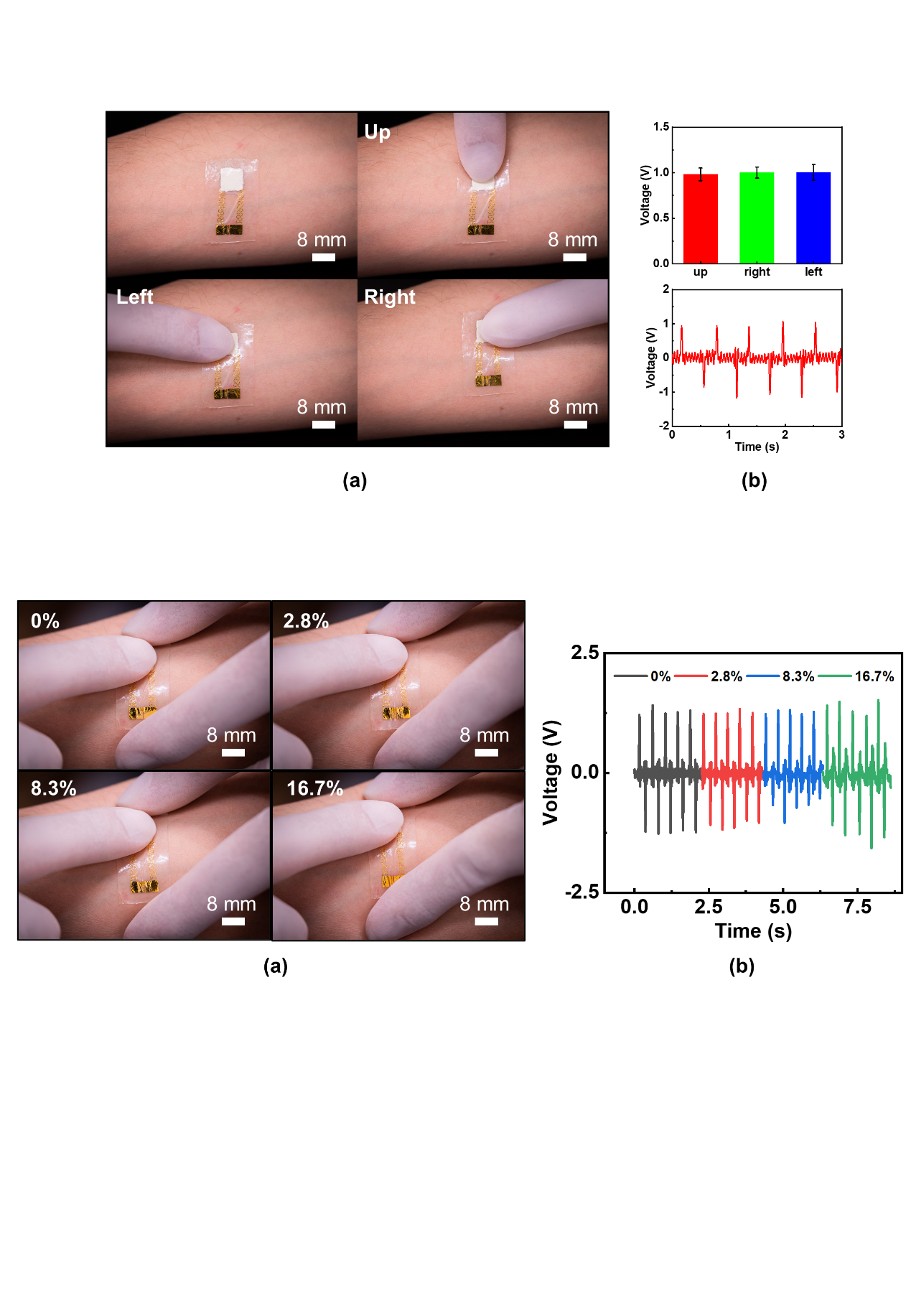


**FIGURE S11.** The electrical response of the 64 mm2 device mounted onto the human skin under a constant stress and frequency of 27.2 kPa and 2 Hz with the three different locations.


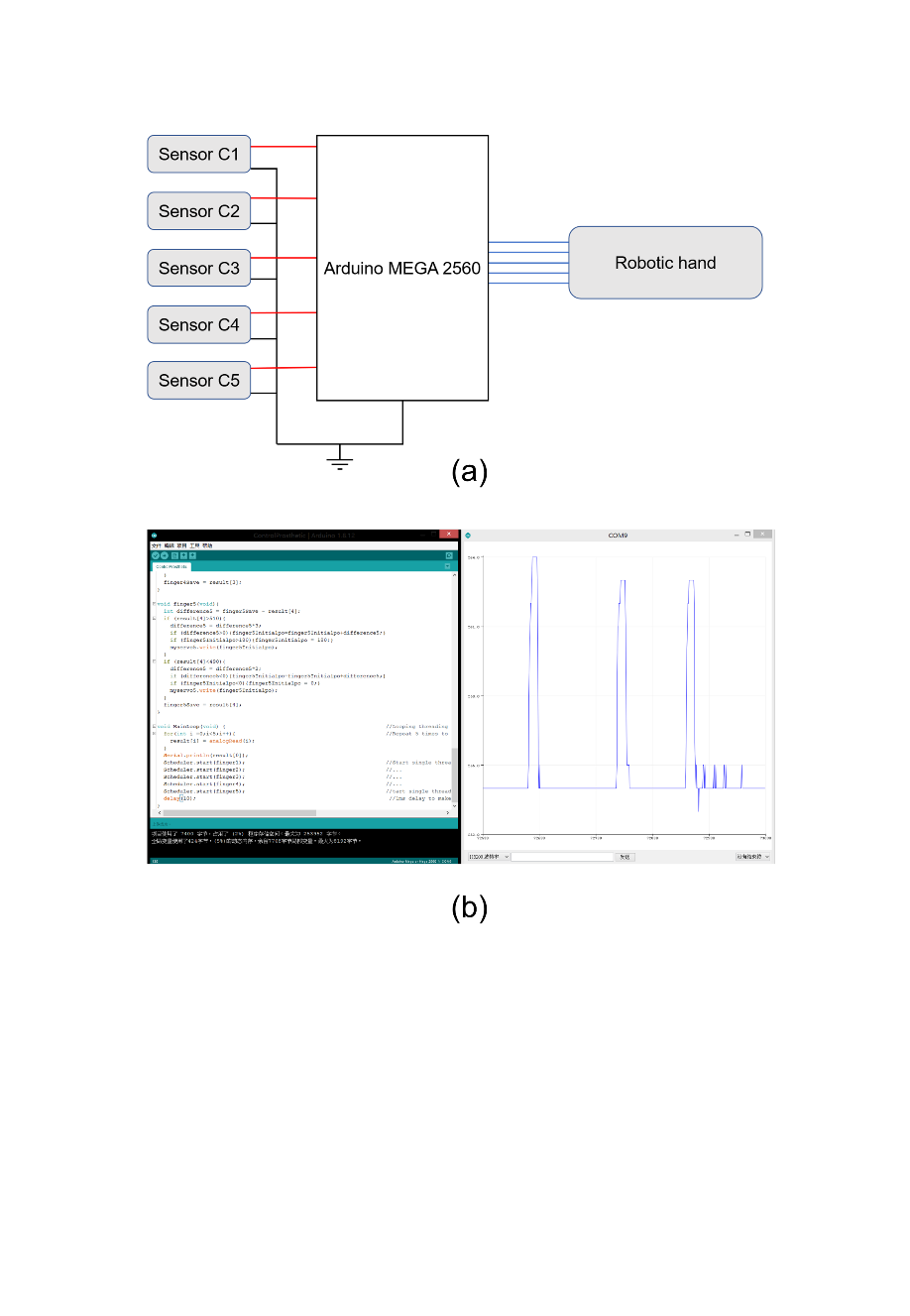


**FIGURE S12.** (**a**) The schematic diagram of the testing circuit for controlling robotic hand. (**b**) The self-developed software interface for controlling robotic hand.


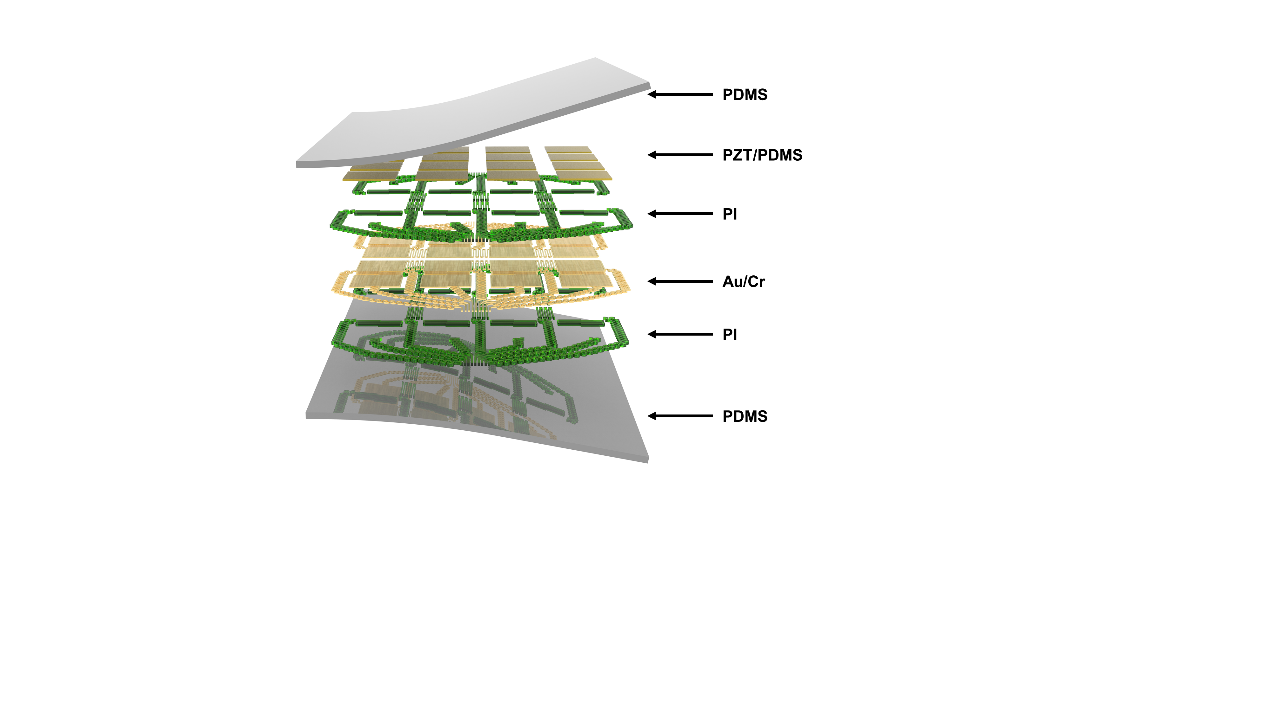


**FIGURE S13.** The exploded-view schematic diagram of the 4×4 array device.


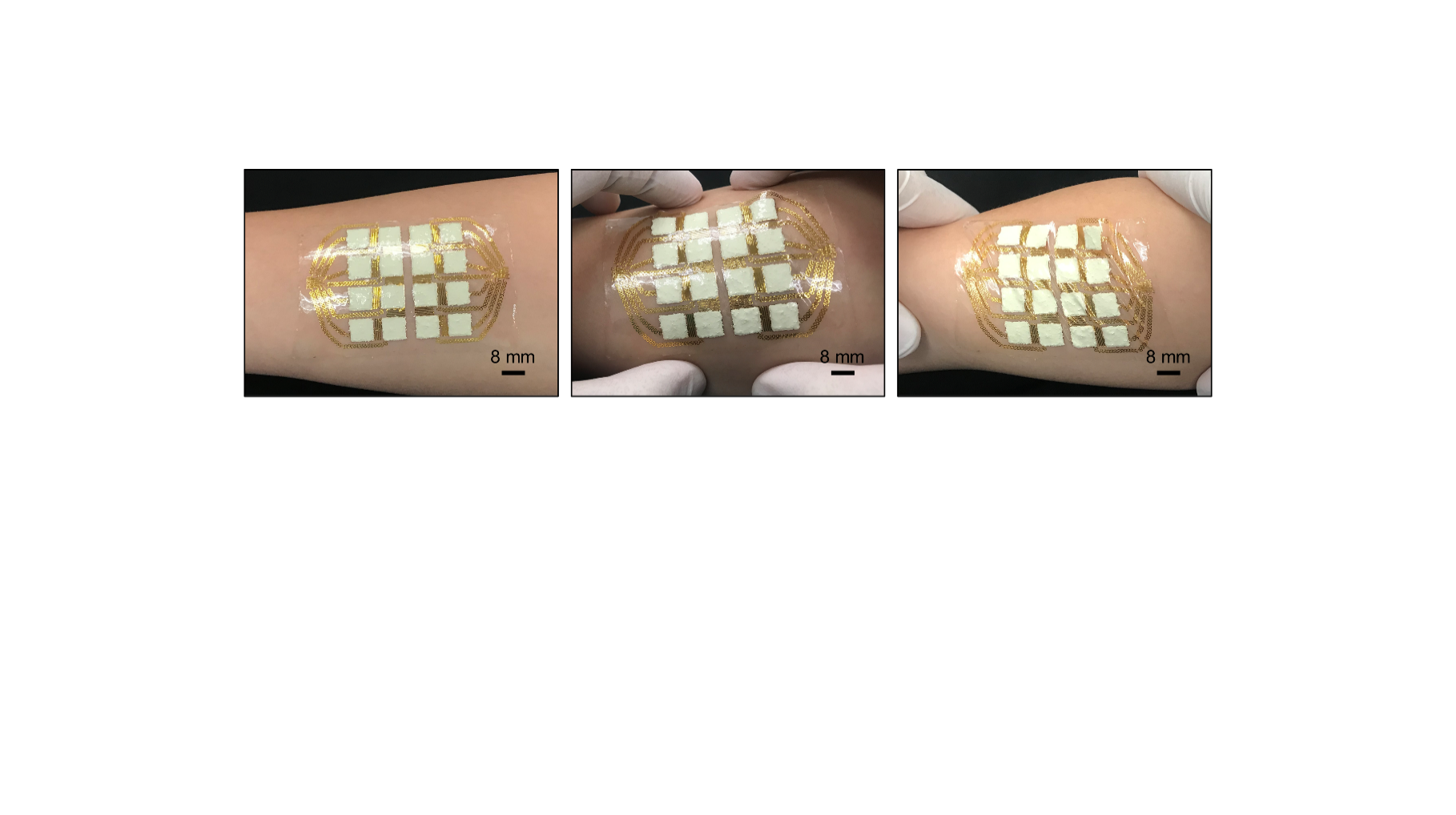


**FIGURE S14.** The optical images of the 4×4 array device mounted onto forearm of an examiner.


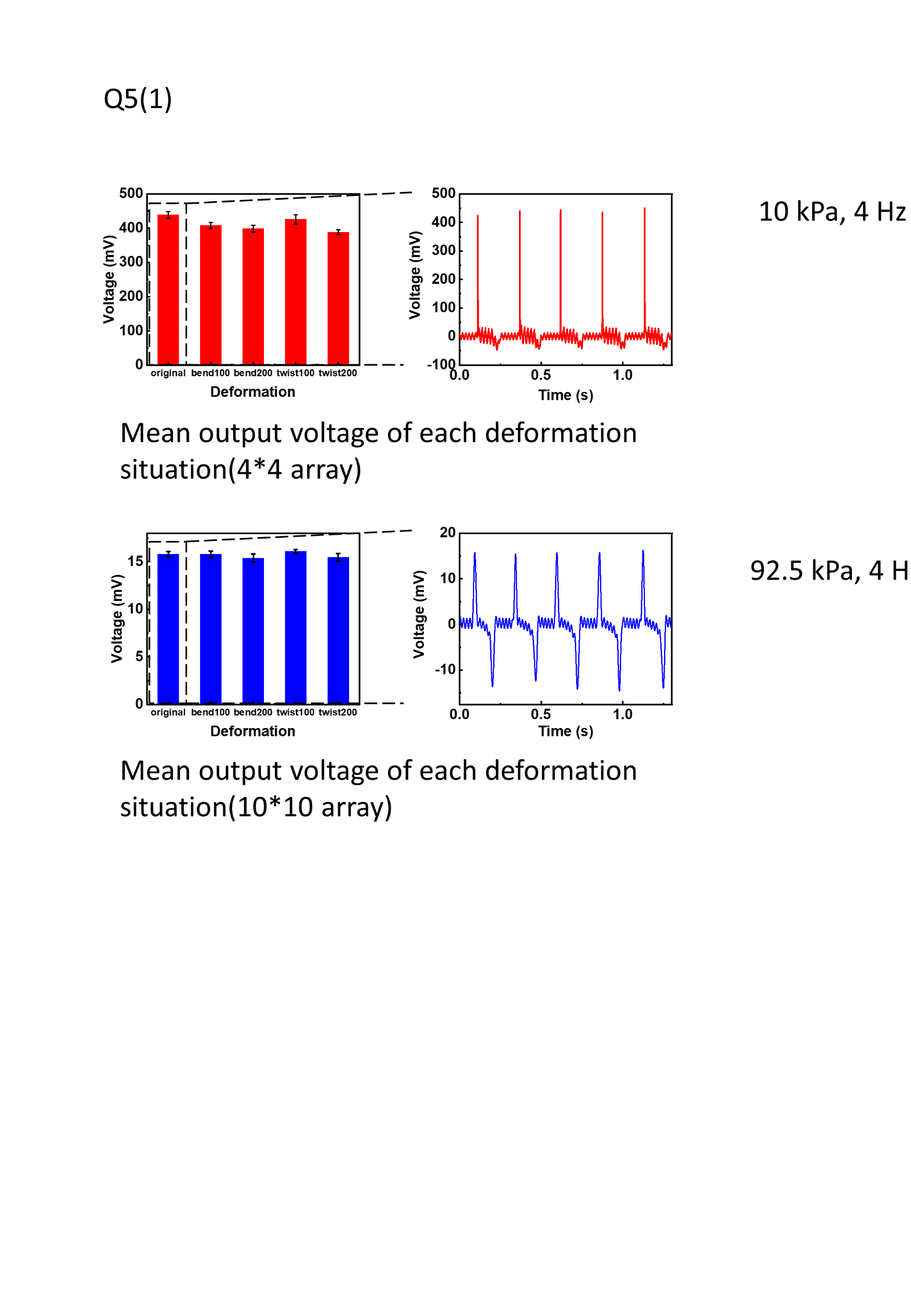


**FIGURE S15.** The electrical signals of the unit of the 4×4 sensor array under original state, bending 100 and 200 cycles, and twisting 100 and 200 cycles at a constant pressure and frequency of 10 kPa and 4 Hz.


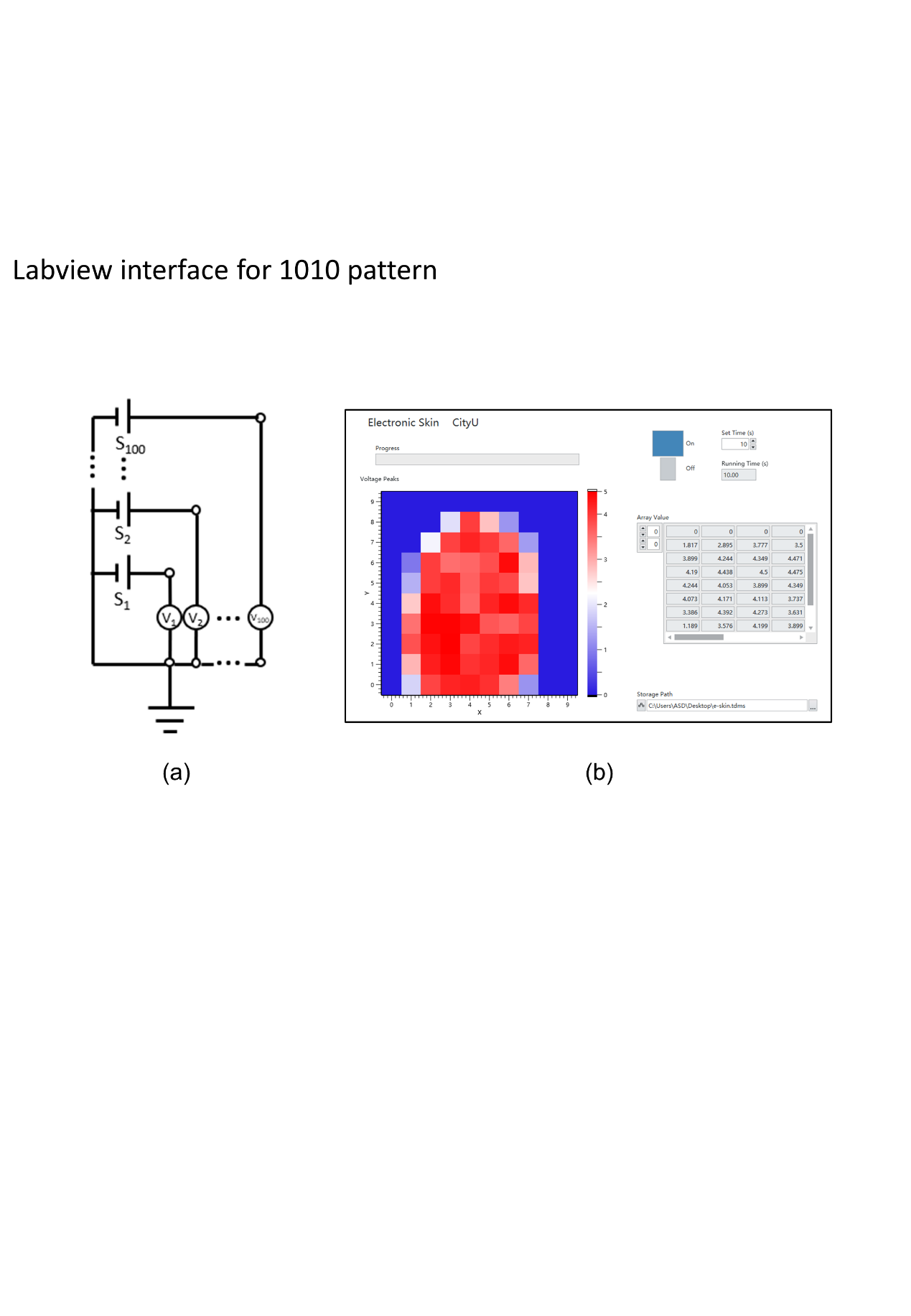


**FIGURE S16.** (**a**) The schematic diagram of the testing circuit for measuring the electrical signal of the multiplexed device. (**b**) The presence of the self-developed software interface as a finger is tapping on the multiplexed device.


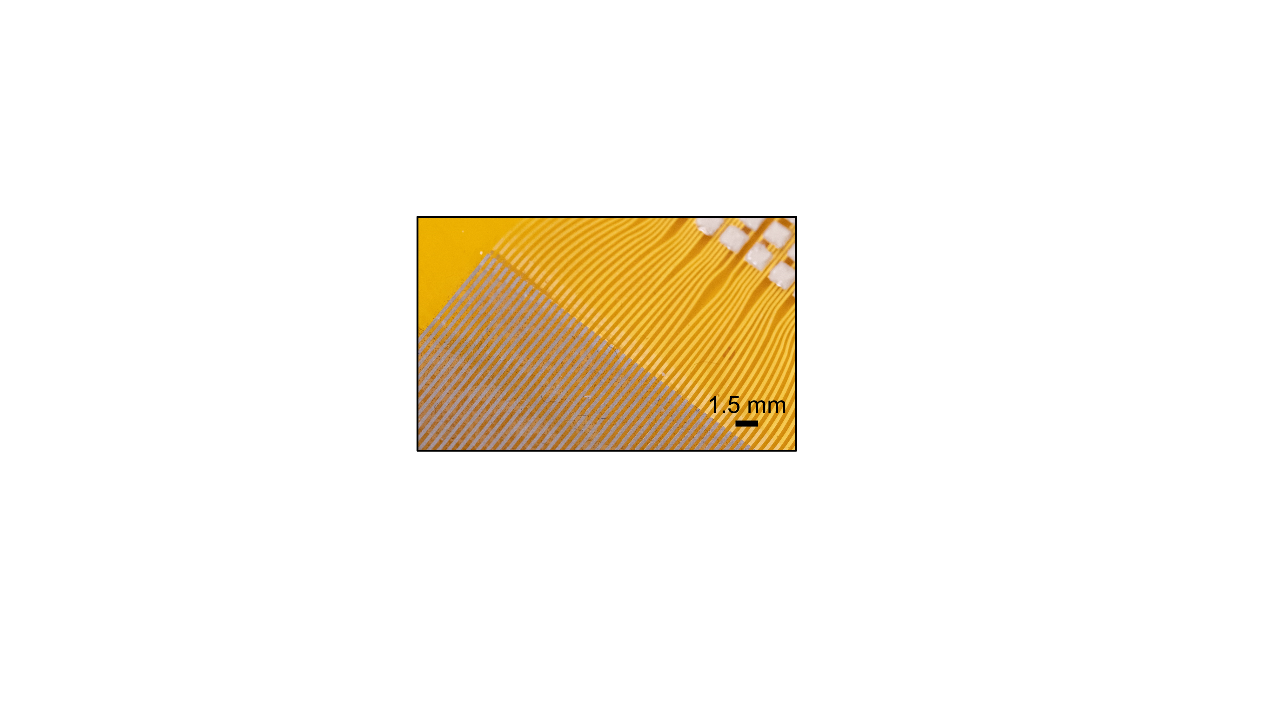


**FIGURE S17.** The enlarged optical image of the connection area between the 10×10 array device and ACF cables.


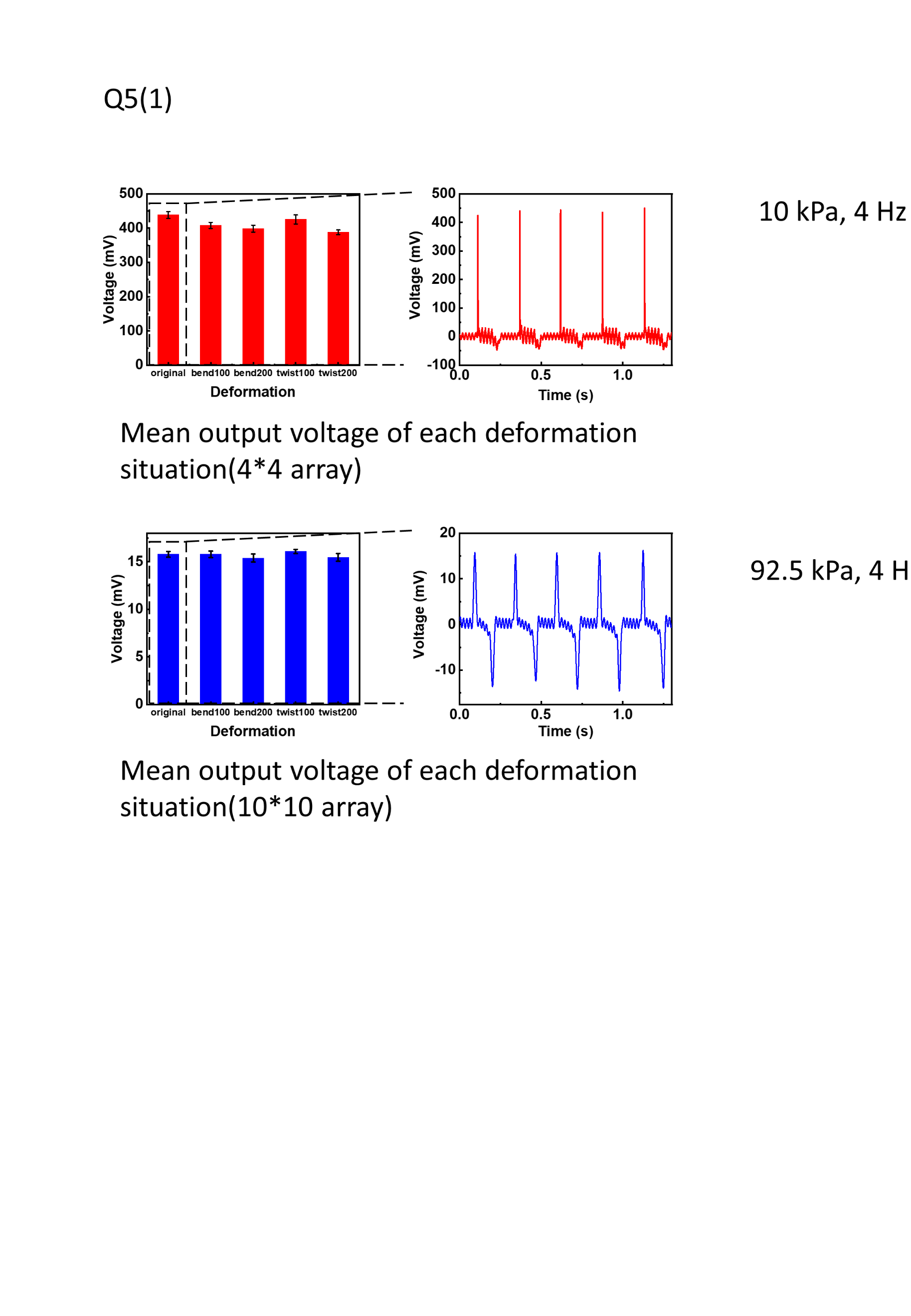


**FIGURE S18.** The electrical signals of the unit of the 10×10 sensor array under original state, bending 100 and 200 cycles, and twisting 100 and 200 cycles at a constant pressure and frequency of 92.5 kPa and 4 Hz.


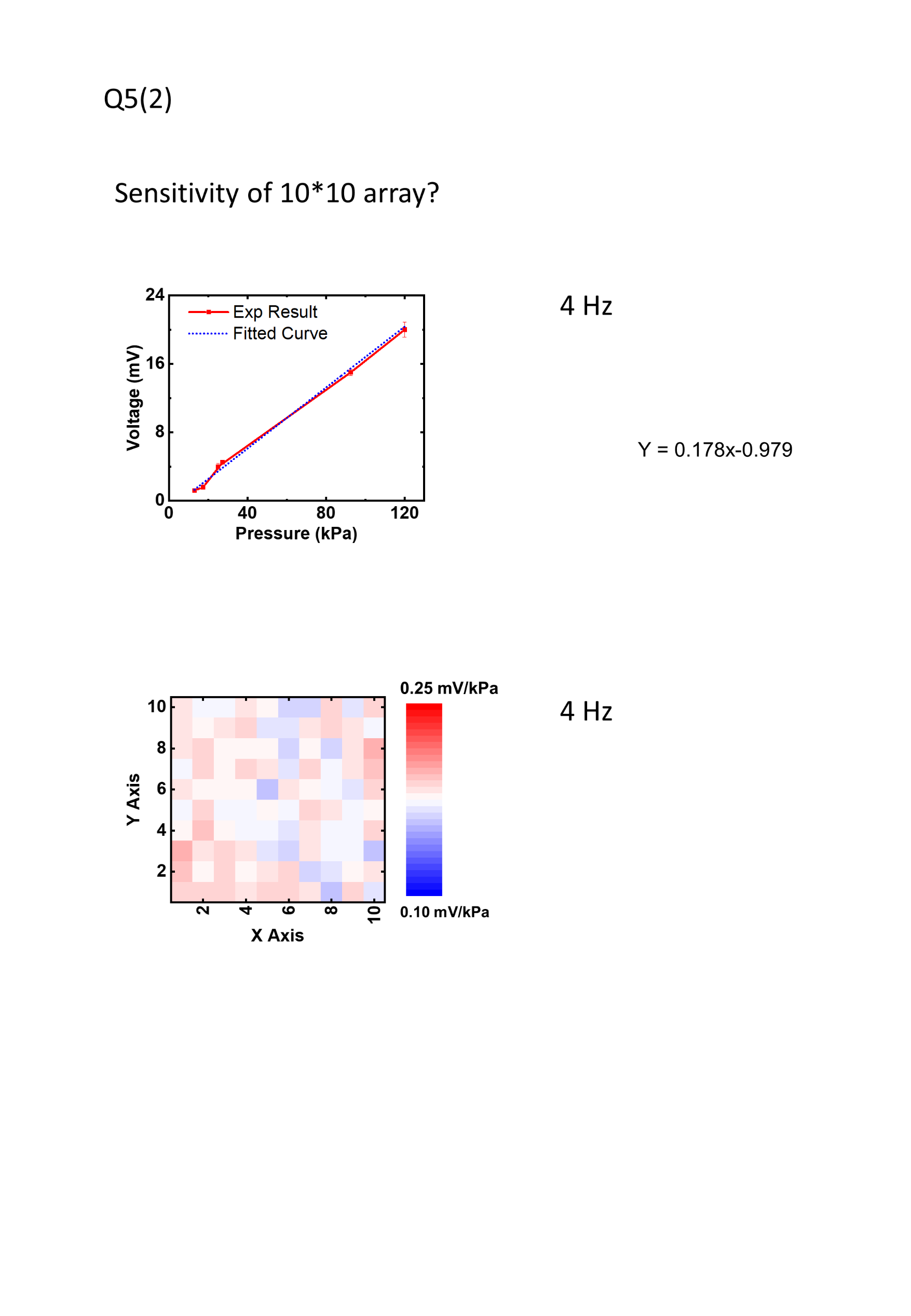


**FIGURE S19.** The electrical signal by a unit of the 10×10 sensor array as a function of pressure, ranging from 12.9 kPa to 120 kPa.


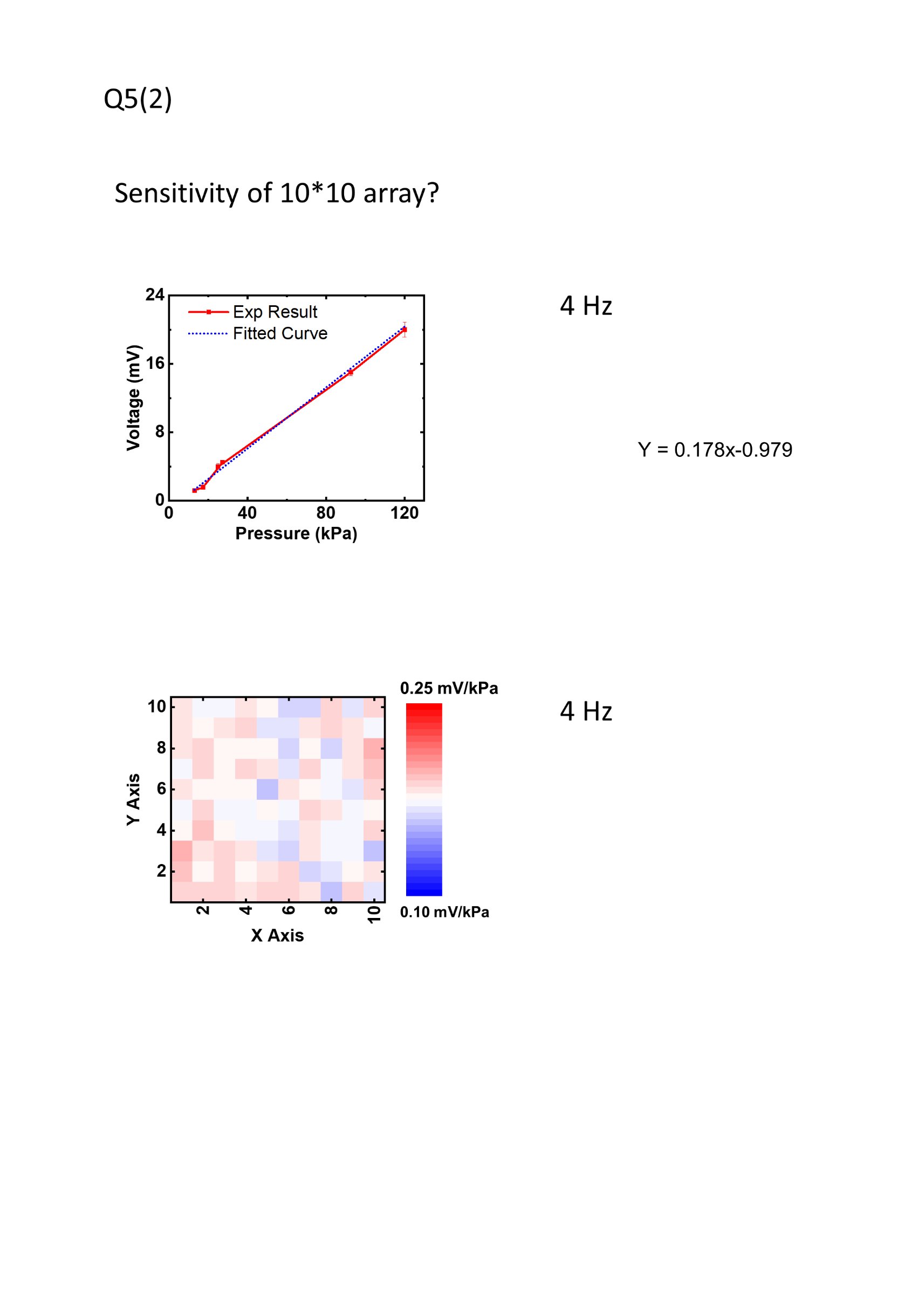


**FIGURE. S20.** The sensitivity of each unit of the 1010 sensor array, ranging from 0.16 mV/kPa to 0.19 mV/kPa.


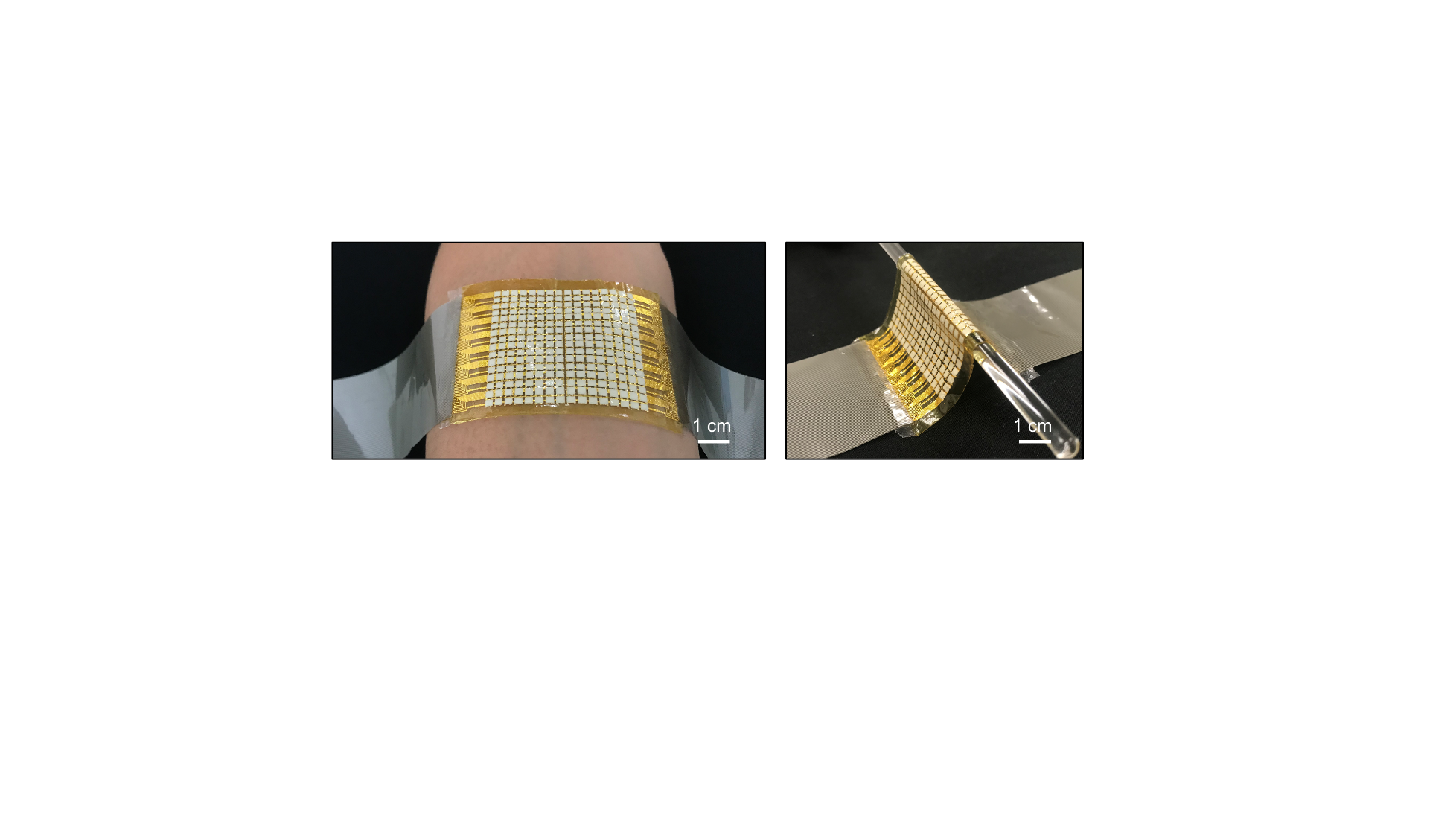


**FIGURE S21.** The optical images of the 1616 array device.
